# Wellbore stability model for argillaceous limestone - claystone thin interbeds under synergistic effect of fluctuating pressure -hydration

**DOI:** 10.1038/s41598-025-23872-5

**Published:** 2025-11-29

**Authors:** Kunhong Lv, Hui Zhang, Xingyu Li, Yuting Zhou, Xingjie Ling, Dexin Ma, Zhi Yang

**Affiliations:** 1 School of Petroleum Engineering, China University of Petroleum (Beijing), Beijing, 102249 China; 2 School of Materials and Chemistry, Southwest University of Science and Technology, Mianyang, 621010 China; 3 China Oilfield Services Ltd., Tianjin, 300459 China

**Keywords:** Thinly interbedded rocks, Fluctuating pressure, Hydration, Wellbore stability model, Collapse pressure, Fracture pressure, Fossil fuels, Engineering

## Abstract

The effect of hydration and fluctuating pressure in drifting conditions presents challenges to wellbore stability, impacting cost savings and safety in drilling operations. This study investigates the stability of thin mudstone-limestone and claystone interlayers in the East Baghdad oil field, introducing strength damage variables influenced by hydration and fluctuating pressure. Utilizing damage mechanics, elasticity, and joint strength theories, and accounting for matrix and weak plane failures, drilling fluid hydration reactions, and fluctuating pressures, a wellbore stability model is established. Key parameters such as wellbore trajectory, weak plane quantity, hydration time on collapse pressure, and tripping speed are examined, assessing stability under combined hydration and pressure effects. The results suggest optimizing wellbore trajectory, particularly the inclination angle, can reduce collapse pressure and increase fracture pressure, thus enhancing operational safety. Weak planes raise collapse pressure, reduce fracture pressure, and limit safe drilling directions, heightening wellbore instability and tripping challenges. Prolonged formation exposure to drilling fluids should be minimized, and fluid density optimized to widen the safe density window. Controlling tripping speed and monitoring wellbore pressure are critical to mitigating instability risks. Field validation confirms the model’s accuracy, aligning predictive outcomes with real conditions and enhancing safe drilling fluid density and tripping speed guidance.

## Introduction

The East Baghdad oil field is located in the northern part of the Mesopotamian Basin, with recoverable reserves up to 11 billion barrels. The lower formations of the East Baghdad oil field are characterized by weak cementation, susceptibility to collapse, developed fractures, instability, irregular infrastructural stresses, and significant variations in stress between well positions, leading to complex issues such as collapses, jams, and leaks, which greatly challenge trajectory control techniques, process applications, and precision control^1^. Ensuring wellbore integrity necessitates enhancing casing centralization and displacement efficiency, thus demanding the deployment of high-stiffness drilling tool assemblies in well sections with substantial trajectory changes, low strength, weak cementation, and fractured formations. During the deployment and retrieval of drilling tools in the field, well collapses and drill jams have frequently occurred, causing great difficulties for drilling safety and operational efficiency^1^. As a principal developmental layer in the East Baghdad oil field, the Sadi formation predominantly features thin interlayers of mudstone-limestone and claystone. In the thin interlayers of mudstone-limestone and claystone formations, wellbore stability issues are more complex, particularly due to differences in water sensitivity, mechanical properties, and pore pressure variations between the two rock types. In the thin interlayers of mudstone-limestone and claystone formations, wellbore stability issues are more complex, particularly due to differences in water sensitivity, mechanical properties, and pore pressure variations between the two rock types. However, the existing models and research primarily focus on single lithology formations or simpler weak plane models, with little attention paid to the specific challenges posed by thin interbedded formations like those in the East Baghdad oil field. These thin interbeds introduce complex interactions between weak planes and hydration effects, which significantly affect wellbore stability.

To address the wellbore stability issue in formations with weak planes, extensive research has been conducted both domestically and internationally. These studies have led to the development of various models that account for shear failure characteristics, stress distribution, and the influence of weak planes. These works can be categorized based on their approach to modeling wellbore stability. The study of wellbore stability began with fundamental efforts to understand shear failure in formations with weak planes. Jaeger^2^ introduced the single weak plane criterion, which allowed for the investigation of shear failure in anisotropic shales. This early model was expanded upon by Aadnoy et al.^3^, who developed a stress model around wellbores, integrating weak plane factors to better account for anisotropy in formations. These pioneering studies laid the foundation for later work that considered a wider range of factors. As research progressed, the impact of drilling fluids and hydration effects on wellbore stability became an important focus. Al-Bazali et al.^4^ were among the first to introduce the hydration effects of drilling fluids on stress distribution, specifically in weak plane-structured formations such as sandstone and shale. This work highlighted the critical role of fluid interactions in stabilizing wellbores. Building on this, Liu et al.^5,6^ extended the model by considering additional environmental factors, such as temperature and seepage, and their effects on weak planes, ultimately leading to a multi-weak-plane model for predicting wellbore collapse pressure. The influence of fractures and cleats on wellbore stability was another key area of research. Qu et al.^7^ developed a model using stress intensity factors to describe the concentration of forces around fractures in coal seams, which contributed to a better understanding of failure mechanisms in fractured formations. Similarly, Lee et al.^8^ introduced a spatial coordinate transformation approach, based on the weak plane strength criterion, to model wellbore stability more accurately in fractured formations. These studies emphasized the importance of considering fractures and weak planes together when assessing wellbore stability. More recent research has focused on developing coupled models that integrate multiple factors influencing wellbore stability. Ma et al.^9^ introduced a mechano-chemical coupled model, which accounts for both mechanical stress and chemical processes, particularly the effects of hydration on the stability of formations with multiple weak planes. Their work demonstrated the necessity of considering the interaction between mechanical forces and geochemical changes when predicting collapse pressure in complex geological formations. Another important development in wellbore stability modeling is the inclusion of time-dependent effects, such as hydration over time and fluid pressure changes. Deng et al.^10^ proposed a force-chemical coupled model that integrates both the weakening effects of drilling fluid hydration and the expansion effects of hydration, with a focus on horizontal shale gas wells. Their model provided insights into the dynamic nature of wellbore collapse pressure as a function of time, marking a shift towards more advanced, time-sensitive predictive models.

Damage constitutive models have become essential tools for predicting the behavior of geological materials under various stress conditions. These models are particularly important for understanding material degradation and its implications for wellbore stability. Wang et al.^11,12^ introduced a damage constitutive model for cemented tailings backfill and rock-encased backfill under uniaxial compression. By using acoustic emission (AE) monitoring, they tracked the damage evolution from crack initiation to failure, integrating both the backfill material and the surrounding rock to improve stability predictions. Zhang et al.^13^ developed a multiscale damage constitutive model to analyze the macro- and microdamage characteristics of gas-bearing coal under loading. Their model focuses on crack initiation and propagation, contributing to the understanding of material degradation in coal, which is critical for stability assessments in coal seam drilling. Duan et al.^14,15^ expanded the understanding of damage evolution in rock masses with weak interlayer zones. Their model considers both loading and unloading stress conditions, emphasizing the interaction between weak interlayer zones and surrounding rock, and capturing the uncoordinated deformation and failure mechanisms. This is crucial for predicting damage in these complex materials. These studies lay the foundation for understanding how weak interlayer zones contribute to damage and failure, which is vital for further research on wellbore stability. Building upon damage models for weak interlayer zones, research has evolved toward developing models specifically aimed at wellbore stability, particularly under the influence of material degradation. Zhang et al.^16^ focused on damage caused by hydration-induced swelling in shale and developed a model to calculate the safe drilling mud density window for shale formations. By employing a Weibull statistical damage model, they captured strength degradation in shale, which is critical for predicting wellbore stability in shale formations under the influence of drilling fluids. Ding et al.^17^ advanced the understanding of wellbore stability by considering the synergistic effects of stress unloading and hydration in shale formations. Their model demonstrates how both unloading-induced damage and hydration weaken shale strength, particularly affecting the wellbore collapse pressure. These studies highlight the feasibility of using damage constitutive models to predict wellbore stability, showing that both material degradation and external factors, such as the influence of drilling fluids, are crucial for understanding and managing wellbore failure.

During the tripping process, fluctuating pressure propagates in the form of elastic waves through the annular drilling fluid, causing changes in the well pressure system and thereby affecting the stress distribution of the surrounding wellbore rock^18,19^. Additionally, due to the physicochemical properties and mechanical characteristics of mudstone-limestone and claystone, under fluctuating pressures, newly initiated and expanding fractures come into contact with drilling fluids, inducing hydration again^20–22^. Literature research shows that existing results mainly focus on the stress unloading and hydration synergy during formation drilling, and the more conventional effects of weak planes and hydration on formation stability after wellbore formation, with little attention to wellbore instability caused by pressure fluctuations during drilling operations citations. Furthermore, research on wellbore stability in weak plane formations primarily focuses on the mechanisms and influencing factors in single lithology formations, with insufficient study on wellbore stability in formations where individual layers are thin and interlayering is frequent^23,24^. Based on the above considerations, this article addresses the instability issues of the wellbore in the thin interlayers of mudstone-limestone and claystone in the East Baghdad oil field, hypothesizing that the claystone thin layers act as a set of low-strength weak planes. Using the Mohr-Coulomb failure criterion and maximum tensile stress theory, combined with laboratory tests of rock mechanical properties to determine the elastic and strength parameters of the rock matrix and weak planes, integrating fluctuating pressure and hydration effects, considering the damage to the rock matrix and weak planes by fluctuating pressure and hydration, a wellbore stability model for mudstone-limestone and claystone thin interlayers has been established. This model explores the variations in wellbore collapse and fracture pressures under changes in hydration time and tripping speed, revealing the mechanical response evolution of wellbore collapse and fracture instability, and thus evaluating the wellbore stability under the combined action of fluctuating pressure and hydration, aiming to guide the optimization design of tripping speed and drilling fluid density, and provide new ideas for safe drilling and efficient development.

## Experimental work

### Preparation of rock specimens

Core samples from the Sadi formation of well M in the East Baghdad oil field show an alternating vertical distribution of black and white (mudstone-limestone and claystone), with uneven layer thicknesses. The mudstone-limestone layers are significantly thicker than those of claystone, displaying characteristics of thin interlayering. In these samples, claystone is regarded as the weak plane within the thin interlayers, and mudstone-limestone as the primary matrix of these interlayers.

**Fig. 1:**
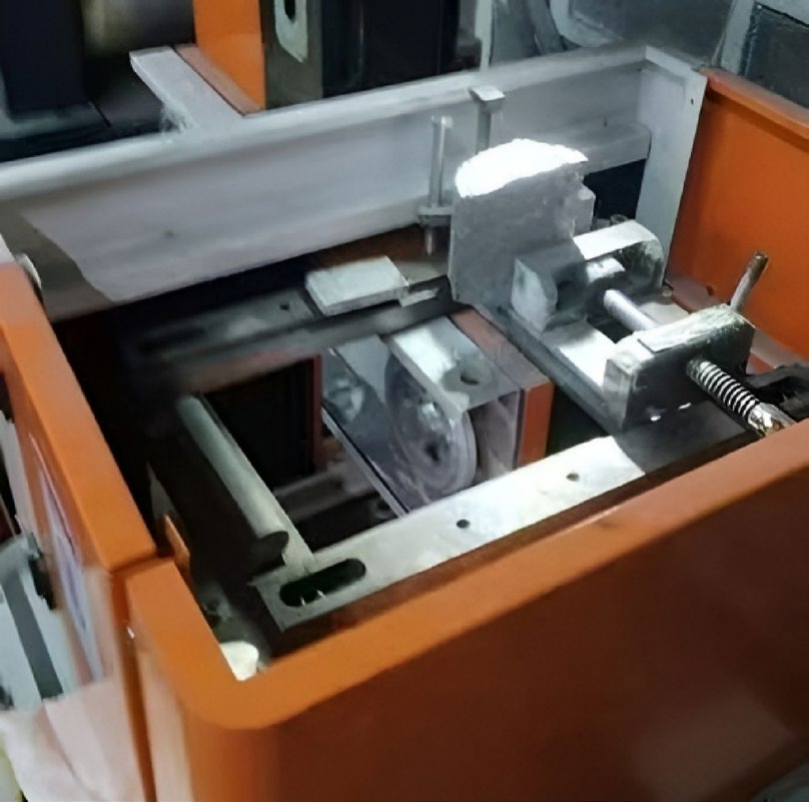
Wire saw.

Specimen preparation was carried out by diamond wire cutting to ensure smooth surfaces and minimal sample damage, thereby improving the reliability and repeatability of subsequent mechanical tests (Fig. 1), a standard procedure widely used in geomechanics and rock mechanics research, with the cutting direction aligned both parallel and perpendicular to the weak planes^25^. This method minimizes mechanical disturbance and thermal effects, ensuring the integrity of the rock samples. Therefore, the reliability of the experimental results is not affected by the preparation technique. Cores parallel to the weak planes, referred to as 90° cores, are used to study the mechanical properties of the weak planes in thin interlayers. Cores perpendicular to the weak planes, referred to as 0° cores, are used to study the mechanical properties of the matrix in thin interlayers.

Mechanical properties of rock matrix and weak planes, after being soaked in drilling fluid, are investigated using triaxial rock mechanics tests. For conducting triaxial rock mechanics tests, cylindrical cores of Φ25 × 50 are used. The cores are required to have a parallelism of ± 0.05 mm between the top and bottom surfaces, and a surface flatness within ± 0.03 mm.

### Triaxial rock mechanics experiments

Triaxial rock mechanics tests are conducted using the ZTR1000 multifunctional experimental system, with displacement-controlled loading at a rate of 0.03mm/min. Confining pressures are set at 0MPa and 10MPa. To avoid randomness in the experimental results, each set of experiments is repeated three times to obtain an average value^1^.

**Fig. 2:**
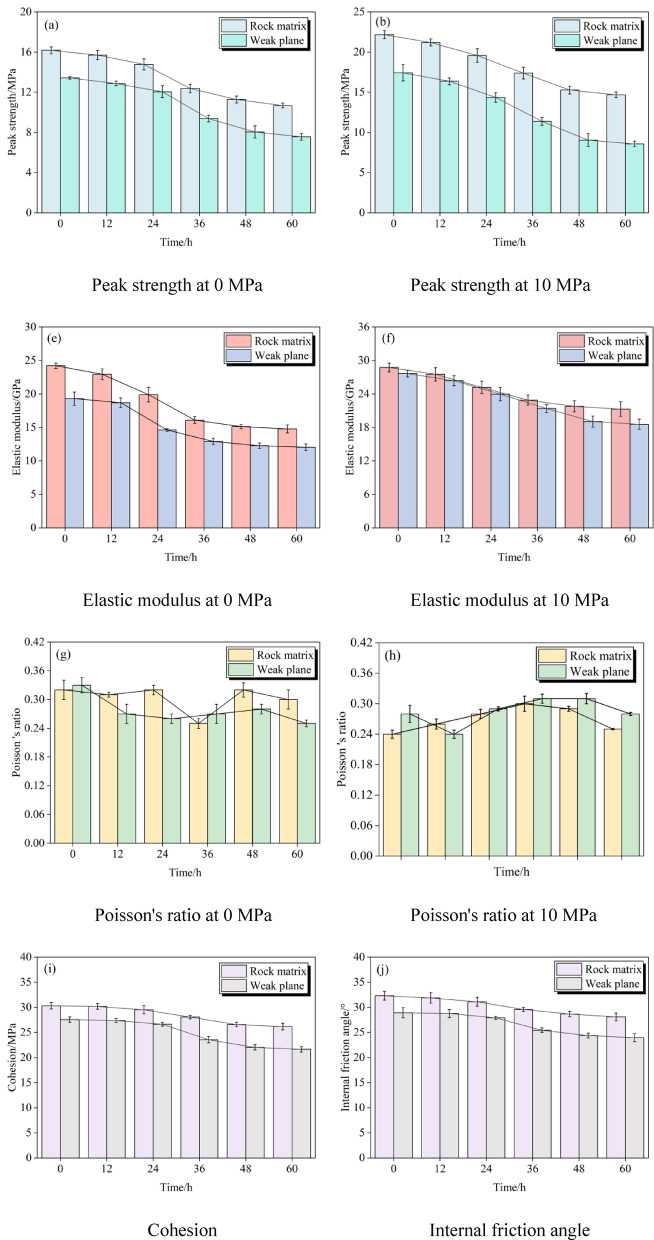
Triaxial mechanical experimental results.

As depicted in Fig. 2, the findings indicate: (1) Hydration effects on material properties: Hydration notably impacts the elastic modulus of the mudstone-limestone and claystone thin interlayer rocks. The elastic modulus decreases over time, with the reduction being more pronounced in the weak planes compared to the rock matrix. However, Poisson’s ratio does not exhibit a clear, consistent trend over time, showing some fluctuations under hydration conditions. (2) Impact of confining pressure: Under 0MPa confining pressure, hydration significantly deteriorates the mechanical properties of the rocks, with both the elastic modulus and Poisson’s ratio showing substantial decreases. In contrast, under 10MPa confining pressure, the rocks exhibit better resistance to deformation and hydration, maintaining relatively stable properties. The elastic modulus at 10MPa confining pressure remains more consistent over time, demonstrating the protective effect of confining pressure. (3) Hydration’s weakening effect on shear strength: Hydration leads to a significant weakening of the shear strength of mudstone-limestone and claystone thin interlayer rocks, which is evident from the noticeable decreases in both internal friction angle and cohesion. The internal friction angle decreases by approximately 5° and cohesion drops by 15% after prolonged hydration. (4) Greater effect on weak planes: The weak planes exhibit more significant degradation under hydration, showing a 25% greater decrease in internal friction angle and cohesion compared to the rock matrix. This suggests that the weak planes are more sensitive to hydration-induced damage, which needs special consideration in engineering design to prevent stability failure.

## Theoretical model

### Circumferential stress distribution

Assume that the discontinuous mudstone-limestone interlayer is an isotropic porous elastic medium. Based on analytical methods derived from Lekhnitskii^26^ and Aadnoy^22^, the wellbore stress in mudstone-limestone formations is composed of in situ stress and stress concentration due to transverse isotropy. Utilizing the superposition principle to combine the aforementioned stress components, the expression for the wellbore stress distribution around mudstone-limestone in a Cartesian coordinate system is derived.

1 $$\left\{ \begin{gathered} \sigma_{x} = \sigma_{x,0} + \sigma_{x,h} = \sigma_{x,0} + 2{\text{Re}} \left[ {\mu_{1}^{2} \phi_{1}^{\prime } \left( {z_{1} } \right) + \mu_{2}^{2} \phi_{2}^{\prime } \left( {z_{2} } \right) + \lambda_{3} \mu_{3}^{2} \phi_{3}^{\prime } \left( {z_{3} } \right)} \right] \hfill \\ \sigma_{y} = \sigma_{y,0} + \sigma_{y,h} = \sigma_{y,0} + 2{\text{Re}} \left[ {\phi_{1}^{\prime } \left( {z_{1} } \right) + \phi_{2}^{\prime } \left( {z_{2} } \right) + \lambda_{3} \phi_{3}^{\prime } \left( {z_{3} } \right)} \right] \hfill \\ \sigma_{z} = \sigma_{z,0} + \sigma_{z,h} = \sigma_{z,0} - \frac{1}{{a_{33} }}\left( {a_{31} \sigma_{x,h} + a_{32} \sigma_{y,h} + a_{34} \tau_{yz,h} + a_{35} \tau_{xz,h} + a_{36} \tau_{xy,h} } \right) \hfill \\ \tau_{xy} = \tau_{xy,0} + \tau_{xy,h} = \tau_{xy,0} - 2{\text{Re}} \left[ {\mu_{1} \phi_{1}^{\prime } \left( {z_{1} } \right) + \mu_{2} \phi_{2}^{\prime } \left( {z_{2} } \right) + \lambda_{3} \mu_{3} \phi_{3}^{\prime } \left( {z_{3} } \right)} \right] \hfill \\ \tau_{xz} = \tau_{xz,0} + \tau_{xz,h} = \tau_{xz,0} + 2{\text{Re}} \left[ {\lambda_{1} \mu_{1} \phi_{1}^{\prime } \left( {z_{1} } \right) + \lambda_{2} \mu_{2} \phi_{2}^{\prime } \left( {z_{2} } \right) + \mu_{3} \phi_{3}^{\prime } \left( {z_{3} } \right)} \right] \hfill \\ \tau_{yz} = \tau_{yz,0} + \tau_{yz,h} = \tau_{yz,0} - 2{\text{Re}} \left[ {\lambda_{1} \phi_{1}^{\prime } \left( {z_{1} } \right) + \lambda_{2} \phi_{2}^{\prime } \left( {z_{2} } \right) + \phi_{3}^{\prime } \left( {z_{3} } \right)} \right] \hfill \\ \end{gathered} \right.$$

where *σ*_*x*_,* σ*_*y*_, *σ*_*z*_ are the wellbore stress tensors along the *x*, *y*, and *z* axes of the mudstone-limestone interlayer, *τ*_*xy*_, *τ*_*xz*_, *τ*_*yz*_ are the wellbore shear stresses along the planes *xy*, *yz*, and *xz* of the mudstone-limestone interlayer, *σ*_*x,*0_, *σ*_*y,*0_, *σ*_*z,*0_ are the wellbore stresses along the *x*, *y*, and z axes under the original in situ stress, *τ*_*xy,*0_, *τ*_*xz,*0_, *τ*_*yz,*0_ are the wellbore shear stresses tangential to the *xy*, *yz*, *xz* planes under the original in situ stress, *σ*_*x,h*_, *σ*_*y,h*_, *σ*_*z,h*_ are the wellbore stresses along the *x*, *y*, *z* axes under transverse isotropy, *τ*_*xy,h*_, *τ*_*xz,h*_, *τ*_*yz,h*_ are the wellbore shear stresses tangential to the *xy*, *yz*, *xz* planes under transverse isotropy, *Re* is the real part of a complex number, *z*_1_, *z*_2_, *z*_3_ are complex variables, *Φ*_1_, *Φ*_2,_ *Φ*_3_ are analytical functions of the transverse isotropy equations, *λ*_1_, *λ*_2_, *λ*_3_ are the ratios of characteristic roots, *μ*_1_, *μ*_2_, *μ*_3_ are the characteristic roots corresponding to the characteristic equations of the strain compatibility.

To facilitate analysis, Cartesian coordinates are converted to wellbore cylindrical coordinates. Based on Bradley’s elastic solution for wellbore stress^27,28^, setting the radial distance to *r* = *R* provides the distribution of wellbore stress within the cylindrical coordinate system.

2 $$\left\{ \begin{gathered} \sigma_{r} = p_{w} - \delta \varphi \left( {p_{w} - p_{p} } \right) \hfill \\ \sigma_{\theta } = - p_{w} + \left( {\sigma_{x,b} + \sigma_{y,b} } \right) - 2\left( {\sigma_{{x,{\text{b}}}} - \sigma_{{y,{\text{b}}}} } \right)\cos (2\theta ) - 4\tau_{xy,b} \sin (2\theta ) + K\left( {p_{w} - p_{p} } \right) \hfill \\ \sigma_{z} = \sigma_{z,b} - 2v\left[ {\left( {\sigma_{x,b} - \sigma_{y,b} } \right)\cos (2\theta ) + 2\tau_{xy,b} \sin (2\theta )} \right] + K\left( {p_{w} - p_{p} } \right) \hfill \\ \tau_{\theta z} = 2\tau_{xy,b} \cos \theta - 2\tau_{xz,b} \sin \theta \hfill \\ \tau_{r\theta } = \tau_{rz} = 0 \hfill \\ \end{gathered} \right.$$

where *σ*_*r*_,*σ*_*θ*_,*σ*_*z*_ are radial, tangential and axial stress of borehole wall in borehole column coordinate, *τ*_*θz*_,*τ*_*rθ*_,*τ*_*rz*_ are the shear stress around the well tangent to *rz*, *θz*, *rθ* planes in wellbore column coordinates, *K* is the coefficient of seepage effect, *p*_*w*_ is the wellbore fluid column pressure, *p*_*p*_ is the formation pore pressure, *φ* is porosity, *θ* is well circumference angle, *v* is static Poisson 's ratio.

3 $$K = \delta \left[ {\frac{\alpha (1 - 2v)}{{1 - v}} - \varphi } \right]$$

where *δ* is wellbore permeability coefficient, *α* is biot’s coefficient.

The degree of damage to the wellbore’s surrounding rock depends on the three principal stresses on the wellbore wall; the radial stress of the wellbore is one of these principal stresses, while the other two principal stresses lie within the *θz* plane. Using the principles of materials mechanics, the three principal stresses on the wellbore can be determined^29^.

4 $$\left\{ \begin{gathered} \sigma_{i} = \sigma_{r} = p_{w} - \delta \varphi \left( {p_{w} - p_{p} } \right) \hfill \\ \sigma_{j} = \frac{{\left( {\sigma_{\theta } + \sigma_{z} } \right)}}{2} + \frac{1}{2}\sqrt {\left( {\sigma_{\theta } - \sigma_{z} } \right)^{2} + 4\tau_{\theta z}^{2} } - \alpha p_{p} \hfill \\ \sigma_{k} = \frac{{\left( {\sigma_{\theta } + \sigma_{z} } \right)}}{2} - \frac{1}{2}\sqrt {\left( {\sigma_{\theta } - \sigma_{z} } \right)^{2} + 4\tau_{\theta z}^{2} } - \alpha p_{p} \hfill \\ \end{gathered} \right.$$

where *σ*_*i*_,* σ*_*j*_, *σ*_*k*_ are the wellbore principal stresses in the *i*, *j*, *k* directions.

By sorting the three principal stresses, the maximum, intermediate, and minimum principal stresses on the wellbore can be identified.

5 $$\left\{ \begin{gathered} \sigma_{1} = \max \left( {\sigma_{i} ,\sigma_{j} ,\sigma_{k} } \right) \hfill \\ \sigma_{3} = \min \left( {\sigma_{i} ,\sigma_{j} ,\sigma_{k} } \right) \hfill \\ \end{gathered} \right.$$

where *σ*_1_,* σ*_3_ are the maximum principal stress and minimum principal stress.

### Damage variable

Under the influence of in situ stress and fluctuating pressure, the failure of mudstone-limestone in drilling fluids can be considered a three-level loading of in situ stress shear stress, hydration tensile stress, and dynamic pressure tensile stress. The overall damage variable can be expressed as^30^:

6 $$D = 1 - \left( {1 - D_{i} } \right)\left( {1 - D_{h} } \right)\left( {1 - D_{s} } \right)$$

where *D* is total damage variable, *D*_*i*_ is damage variable under in-situ stress load, *D*_*h*_ is damage variable under hydration, *D*_*s*_ is damage variable under fluctuating pressure.

Assuming that the initial damage to the wellbore surrounding rock is zero before the commencement of tripping operations, then:

7 $$D = 1 - \left( {1 - D_{h} } \right)\left( {1 - D_{s} } \right)$$

### Wellbore instability judgment

Applying the Mohr-Coulomb criterion with consideration of the effective stress principle, the shear strength failure criterion can be stated as:

8 $$\sigma_{1} - \alpha p_{p} = \frac{{2c_{0} \cos \varphi }}{1 - \sin \varphi } + \frac{1 + \sin \varphi }{{1 - \sin \varphi }}\left( {\sigma_{3} - \alpha p_{p} } \right)$$

where *c*_0_ is cohesion.

Formation fracturing occurs when the circumferential stress exerted on the rock by the overly dense drilling fluid in the well reaches the rock’s tensile strength. The criterion for tensile strength failure is:

9 $$\sigma_{3} - \alpha p_{p} = - \sigma_{t}$$

where *σ*_*t*_ is the rock’s tensile strength.

Evaluating the rock’s tensile strength based on the stress state of the wellbore:

10 $$\sigma_{t} = \frac{1}{2}\left( {\sigma_{\theta } + \sigma_{z} } \right) - \frac{1}{2}\sqrt {\left( {\sigma_{\theta } - \sigma_{z} } \right)^{2} + 4\tau_{\theta z}^{2} } - \alpha p_{{\text{p}}}$$

Establishing a rock damage constitutive equation based on Lemaitre’s strain equivalence theory^31^:

11 $$\left[ {\sigma^{ * } } \right] = [\sigma ]/(1 - D)$$

where [σ^*^] is dffective stress matrix, [*σ*] is nominal stress matrix.

The revised principle of effective stress considering damage is^32^:

12 $$\sigma_{m}^{*} = \sigma_{m} /\left( {1 - D} \right)$$

where *σ*_*m*_ is the component of ground stress tensor, *σ*_m_^*^ is the effective stress considering damage.

Considering the rock interfaces of the mudstone-limestone interlayer as weak planes, based on the single weak plane criterion, a function *f*_*c*_ is defined to solve and determine whether shear failure occurs along the rock matrix or the weak planes around the wellbore:

13 $$f_{c} \left( {p_{c} } \right) = \left\{ \begin{gathered} 2c_{\omega } \left( {1 - D} \right)\cos \left[ {\left( {1 - D} \right)\varphi _{\omega } } \right] + \sin \left[ {\left( {1 - D} \right)\varphi _{\omega } } \right]\left( {\sigma _{1}^{i} + \sigma _{3}^{i} - 2\alpha p_{p} } \right) - \left( {\sigma _{1}^{i} - \sigma _{3}^{i} } \right){\text{ }}\left( {\beta _{1} \leqslant \beta \leqslant \beta _{2} } \right) \hfill \\ 2c_{0} \left( {1 - D} \right)\cos \left[ {\left( {1 - D} \right)\varphi _{0} } \right] + \sin \left[ {\left( {1 - D} \right)\varphi _{\omega } } \right]\left( {\sigma _{1}^{i} + \sigma _{3}^{i} - 2\alpha p_{p} } \right) - \left( {\sigma _{1}^{i} - \sigma _{3}^{i} } \right){\text{ }}\left( {\beta < \beta _{1} {\text{ }}or{\text{ }}\beta > \beta _{2} } \right) \hfill \\ \end{gathered} \right.$$

where *φ*_0_ is internal friction angle of rock matrix, *c*_*w*_ is weak plane cohesion, *φ*_*w*_ is weak plane internal friction angle, *p*_*c*_ is collapse pressure, *f*_*c*_*(p*_*c*_*)* is nonlinear function of wellbore collapse.

If *f*_*c*_ < 0, the wellbore surrounding rock will undergo shear failure; if *f*_*c*_ = 0, the wellbore surrounding rock is in a state of limit equilibrium; if *f*_*c*_ > 0, there will be no shear failure in the wellbore surrounding rock.

Define a function *f*_*f*_ to determine whether tensile failure occurs in the surrounding strata of the wellbore:

14 $$f_{f} \left( {p_{f} } \right) = \left\{ \begin{gathered} \sigma_{i}^{*} - \alpha p_{p} + \sigma_{t} = \sigma_{i} /\left( {1 - D} \right) - \alpha p_{p} + \sigma_{t} \hfill \\ \sigma_{i}^{*} - \alpha p_{p} + \sigma_{t}^{*} = \sigma_{i} /\left( {1 - D} \right) - \alpha p_{p} + \sigma_{t} \hfill \\ \end{gathered} \right.$$

where *σ*_t_^*^ is Weak plane tensile strength, *f*_*f*_*(p*_*f*_*)* is nonlinear function of wellbore fracture, *p*_*f*_ is fracture pressure (MPa).

In Eq. 14, a function *f*_*f*_(*p*_*f*_) is defined to account for key factors that influence shear failure, including cohesion, friction angle, pore pressure, and collapse pressure. The function is expressed as:

15 $$f_{c} (p_{c} ) = f\left( {\sigma_{1} ,\sigma_{2} ,c_{0} ,\varphi_{0} ,c_{w} ,\varphi_{w} ,p_{p} ,p_{c} } \right)$$

If *f*_*f*_ < 0, the wellbore surrounding rock will undergo tensile failure; if *f*_*f*_ = 0, the wellbore surrounding rock is in a state of limit equilibrium; if *f*_*f*_ > 0, there will be no tensile failure in the wellbore surrounding rock.

### Model solving process

**Fig. 3:**
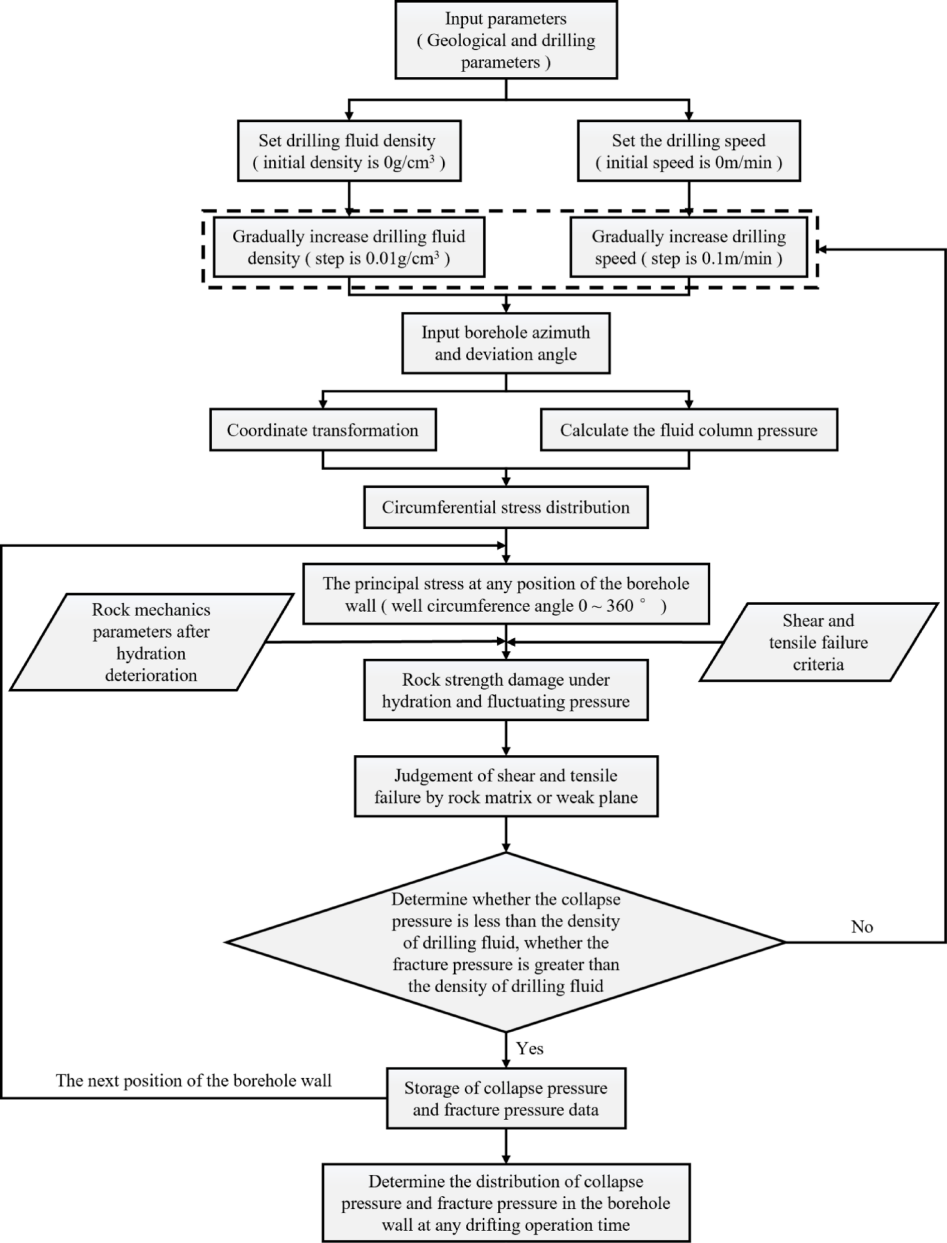
Calculation flow chart of fluctuating pressure-hydration wellbore stability model.

Given the complexity of solving for wellbore stress and the iterative process for collapse and fracture pressures, MATLAB computational platform is used for programming and simulation calculations, with the specific process illustrated in Fig. 3. Initially, input parameters are determined based on completed drilling data, setting the initial density of the drilling fluid and the initial tripping speed. The computational step size is divided according to accuracy requirements to solve for wellbore stresses and principal stresses in the fluctuating pressure-hydration wellbore stability model; Subsequently, based on damage theory and hydration degradation test results, the rock strength damage variables under hydration and fluctuating pressure are identified, clarifying the type of wellbore surrounding rock damage, and calculating the collapse and fracture pressures at a certain depth under the combined effect of fluctuating pressure and hydration; Finally, by cyclically iterating the solutions for collapse and fracture pressures at different positions of the wellbore, the effects of various factors on the drilling fluid density window are determined, and the optimal values or ranges for drilling fluid density and tripping speed are recommended.

## Synergistic effect analysis of fluctuating pressure - hydration

**Fig. 4:**
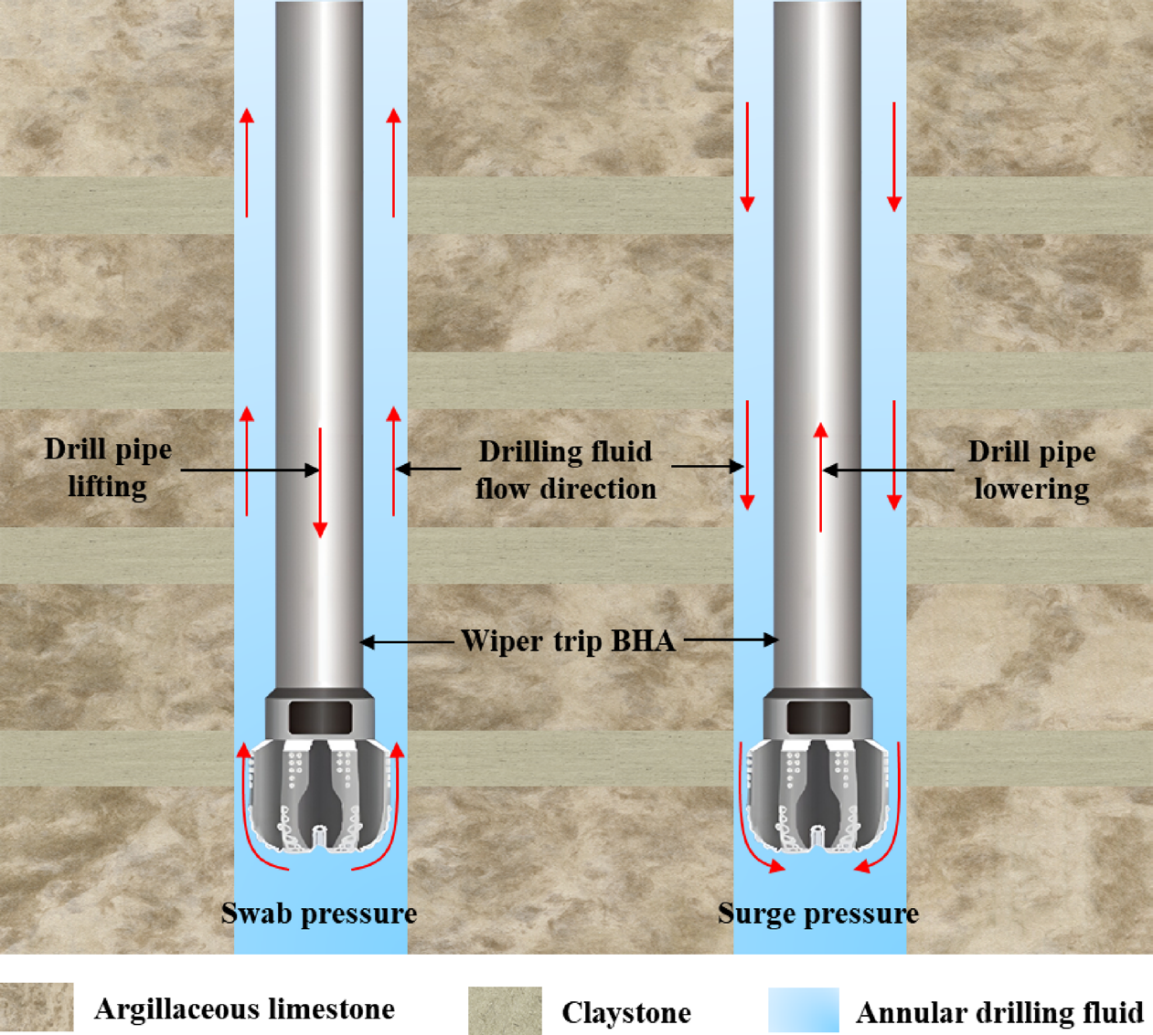
Fluctuating pressure schematic diagram.

As shown in Fig. 4, during the tripping in and out of the BHA, due to the displacement effect of the lower BHA assembly and centralizers, and the adhesive action on the surface of the drill string, drilling fluid flows upward or downward in the annulus, causing pressure changes within the wellbore. Lowering the drill string generates additional surge pressure, while raising it produces additional swab pressure, collectively referred to as fluctuating pressure^33^. Dynamic pressure propagates as elastic waves through the drilling fluid in the annulus, altering the well’s internal pressure system and increasing the risk of wellbore instability on top of the existing deterioration caused by hydration effects on the rock. Swab pressure resulting from raising the drill string reduces bottom-hole pressure, potentially leading to collapse instability. Surge pressure from lowering the drill string leads to stress concentration around weak planes like bedding and fractures in the wellbore wall rock, ultimately causing instability and rupture. Therefore, conducting research on wellbore stability under the combined effects of fluctuating pressure and hydration is of significant importance for the safety of drilling operations.

### Establishment of fluctuating pressure calculation model

Calculation of dynamic pressure primarily includes steady-state and transient methods. The steady-state method offers greater operability in the field compared to the transient method, and its results are more conservative, which is more beneficial for safe on-site construction. Due to the presence of the static shear force of the drilling fluid, the drill string needs to overcome this static shear force during movement, causing the surrounding drilling fluid to move in the same direction, thereby generating dynamic pressure^34,35^:

16 $${\Delta }p_{1} = \frac{{4\tau_{s} L}}{{r_{w} - r_{{{\text{co}}}} }}$$

where *∆p*_1_ is fluctuating pressure caused by static shear force of drilling fluid, *τ*_*s*_ is static shear force of drilling fluid, *L* is the drill string length, *r*_*w*_ is the wellbore diameter, *r*_*co*_ is the outer diameter of the drill string.

The viscous resistance of the drilling fluid causes the surrounding drilling fluid to move in the same direction as the drill string. Considering changes in the annular gap caused by components such as centralizers, the pressure drop caused by the centralizers in the annular drilling fluid is treated as local head loss, resulting in parameters that reflect the impact of centralizers on annular pressure loss^36^:

17 $$f_{p} = 1 + \frac{{r_{w} - d_{f} }}{{f\left( {L_{2} + L_{1} } \right)}}\frac{{d_{f}^{2} - r_{co}^{2} }}{{r_{w}^{2} - d_{f}^{2} }}\left( {\frac{{r_{w}^{2} - r_{co}^{2} }}{{r_{w}^{2} - d_{f}^{2} }} - \frac{1}{2}} \right)$$

The dynamic pressure caused by the viscosity of the drilling fluid due to the centralizer is as follows:

18 $${\Delta }p_{2}^{\prime } = f_{p} {\Delta }p_{2}$$

where *f*_*p*_ is the parameter variables of the influence of centralizer on annular pressure loss, *f* is annular friction coefficient, *L*_1_ is length of centralizer, *L*_2_ is casing length between centralizers, *d*_*f*_ is outer diameter of centralizer, ∆*p*_2_´is the fluctuating pressure caused by the viscous force after the centralizer is considered, *∆p*_2_ is the fluctuating pressure generated by viscous force.

The dynamic pressure generated by viscous forces is:

19 $$\Delta p_{2} = \frac{{0.196f_{m} \rho_{m} v_{m}^{2} L}}{{\left( {r_{w} - r_{co} } \right)}}$$

where *f*_*m*_ is friction coefficient, *ρ*_*m*_ is drilling fluid density, *v*_*m*_ is average flow rate of drilling fluid, equivalent to the string up and down speed.

In the process of raising and lowering the drill string, the acceleration of the drill string alters the momentum of the drilling fluid, which results in the following dynamic pressure^35^:

20 $${\Delta }p_{3} = \frac{{\rho_{m} r_{co}^{2} La}}{{r_{w}^{2} - r_{co}^{2} }}$$

where *∆p*_3_ is the fluctuating pressure caused by the inertial force of the pipe string, *a* is acceleration of BHA lowering.

Consequently, the total dynamic pressure resulting from the drill string’s movement in the drilling fluid is:

21 $$\Delta p = \Delta p_{1} + {\Delta }p_{2}^{\prime } + \Delta p_{3}$$

where *∆p* is the total fluctuating pressure.

### Borehole wall damage under fluctuating pressure

**Fig. 5:**
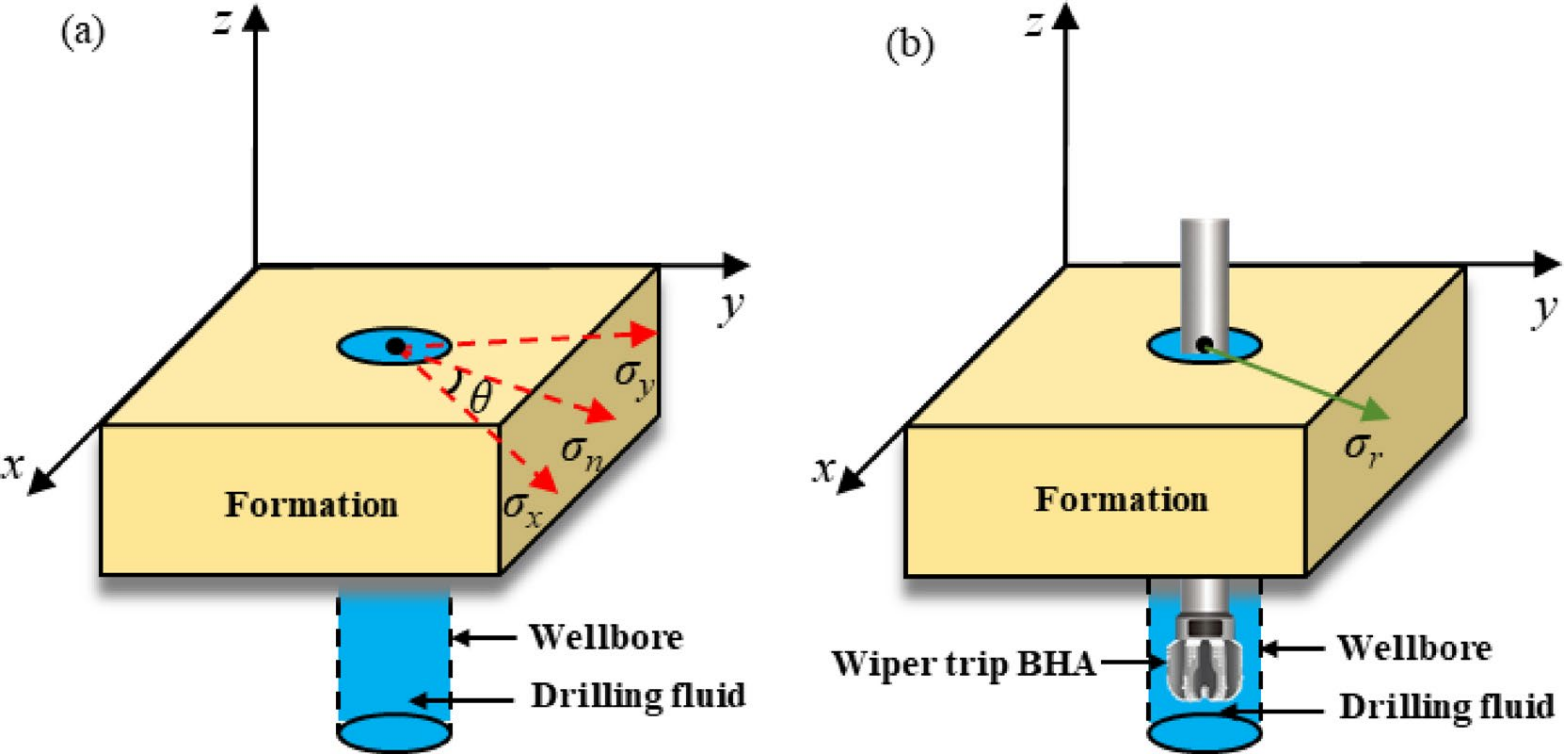
The schematic diagram of circumferential stress under the action of fluctuating pressure: (**a**) before drifting condition; (**b**) after drifting condition.

Before tripping the drill string down and up, when the surrounding rock and annular fluid are in a static state, the stress state of the surrounding rock is mainly controlled by the in-situ stress, with the distribution of in situ stress shown in Fig. 5. During the lowering and raising of the drill string, dynamic pressure affects the wellbore pressure and acts directly on the radial direction of the well wall. This change further influences the tangential and axial stresses. Therefore, axial and circumferential stresses can be disregarded, and the changes in radial stress during the movement of the drill string can represent the variations in dynamic pressure stress.

22 $${\Delta }\sigma = \sigma_{n} - \sigma_{r} = \sigma_{n} - \left[ {p_{{\text{w}}} - \delta \phi \left( {p_{{\text{w}}} - p_{{\text{p}}} } \right)} \right]$$

23 $$\sigma_{n} = \pm \left( {\sigma_{y} \sin \theta + \sigma_{x} \cos \theta } \right)$$

where *∆σ* is stress change under fluctuating pressure, *σ*_*n*_ is the stress component of in-situ stress in the direction of fluctuating pressure, taking a positive value when the drill string is tripped in, and a negative value when tripped out.

The magnitude of the damage variable under dynamic pressure depends on the angle of the weak planes, the direction of the dynamic pressure, the angle between the direction of the dynamic pressure and the weak planes, the angle between the direction of the dynamic pressure and the principal stress direction, the changes in stress under pressure fluctuations, and the original wellbore stress. Based on the generalized Hooke’s law^37^ and combining previous mechanical experimental results, the damage variable under dynamic pressure, using the modulus of elasticity method based on the strain equivalence assumption, can be expressed as:

24 $$D_{s} \left( {\sigma_{x,y,z} ,\sigma_{n} ,{\Delta }\sigma ,\beta_{mw} ,\beta_{sw} } \right) = 1 - \frac{{E_{s} }}{E}$$

where *β*_*mw*_ is the angle between the maximum principal stress and the weak plane, *β*_*sw*_ is the angle between the principal stress and the direction of fluctuating pressure, *E*_*s*_ is the elastic modulus of the rock sample under fluctuating pressure, *E* is the elastic modulus of the undisturbed rock sample.

### Borehole wall damage under hydration

Using hydration-induced strain as the fundamental strength parameter, the hydration damage variable for rock samples is defined as^32,38^:

25 $$D_{h} = 1 - \exp \left[ { - \left( {\frac{{\varepsilon_{h} }}{{\varepsilon_{h,0} }}} \right)^{n} } \right] \,$$

where *ε*_*h*_ is hydration volume strain, *ε*_*h,*0_ is average hydration volume strain, *n* is hydration damage coefficient.

The change in hydration volume strain over time can be expressed as:

26 $$\frac{{\partial \varepsilon_{h} }}{\partial t} = \frac{\alpha - 1}{{G_{K} }}\mathop \sum \limits_{k} \omega_{k} \frac{{\partial \mu_{k} }}{{\partial c_{k} }}\frac{{\partial c_{k} }}{\partial t} \,$$

where *G*_*K*_ is bulk modulus of the rock sample, *ω*_*k*_ is hydration expansion coefficient related to the chemical potential of component* k*, *μ*_*k*_ is chemical potential of component *k* within the pore fluid, *c*_*k*_ is molar concentration of solute component* k*, *t* is hydration time, *k* is solute component number.

If the drilling fluid contains only one type of solute associated with hydration expansion, the concentration-related damage coefficient is^39^:

27 $$\omega_{1} = \frac{\alpha - 1}{{G_{K} }}\omega_{k0} \frac{{\partial \mu_{k} }}{\partial c} \,$$

where *ω*_1_ is damage coefficient related to concentration, *ω*_*k0*_ is hydration expansion coefficient related to the chemical potential of a single component, *c* is molar concentration of solutes.

By combining Eq. (25) and (26), we obtain:

28 $$D_{h} = 1 - e^{{( - \frac{{c\omega_{1} }}{{\varepsilon_{h,0} }})^{n} }}$$

The damage variable of rock samples due to hydration is defined as:

29 $$D_{h} = 1 - \exp \left[ { - \left( {\frac{\alpha - 1}{{G_{K} }} \cdot \frac{{\partial \mu_{k} }}{\partial c} \cdot \frac{{c\omega_{k0} }}{{\varepsilon_{h,0} }}} \right)^{n} } \right] \,$$

## Wellbore stability analysis

The target layer for well M in the East Baghdad oil field is the Sadi formation, consisting of mudstone-limestone and claystone thin interlayers. Based on the model constructed in this study, wellbore stability at a depth of 3725 meters is analyzed. The basic parameters are shown in Table 1, and the effects of well trajectory, the number of weak planes, hydration time, and tripping speed on collapse and fracture pressures are discussed.

**Table 1:** Basic parameters.

Parameter name	Parameter value	Parameter unit
Well depth	3725.00	m
Wellbore radius	0.12	m
Overburden pressure equivalent density	2.24	g/cm^3^
Maximum horizontal in situ stress equivalent density	2.41	g/cm^3^
Minimum horizontal in situ stress equivalent density	1.87	g/cm^3^
Pore pressure equivalent density	1.13	g/cm^3^
Liquid column pressure equivalent density	1.24	g/cm^3^
Effective stress coefficient	0.80	–
Porosity	0.07	–
Poisson’s ratio	0.25	–
Matrix elastic modulus		GPa
Matrix internal friction angle	32.53	°
Matrix cohesion	21.70	MPa
Weak plane internal friction angle	25.37	°
Weak plane cohesion	16.32	MPa
Tensile strength	2.00	MPa

### Case analysis

Taking well M as an example, with an initial drilling fluid density of 1.55g/cm^3^, severe resistance and high torque were encountered during reaming, and the circulating drilling fluid contained numerous flat and plate-like fragments approximately 1~2 cm in size, confirming that wellbore instability during drilling was controlled by weak planes. As the drilling fluid density was gradually increased, although collapses were still evident, the size of the fragments decreased, indicating an improvement in wellbore stability. When the drilling fluid density was increased to 1.79g/cm^3^, the drilling assembly experienced no resistance or sticking during tripping in and out, as evidenced by the lack of any jamming issues.

**Fig. 6:**
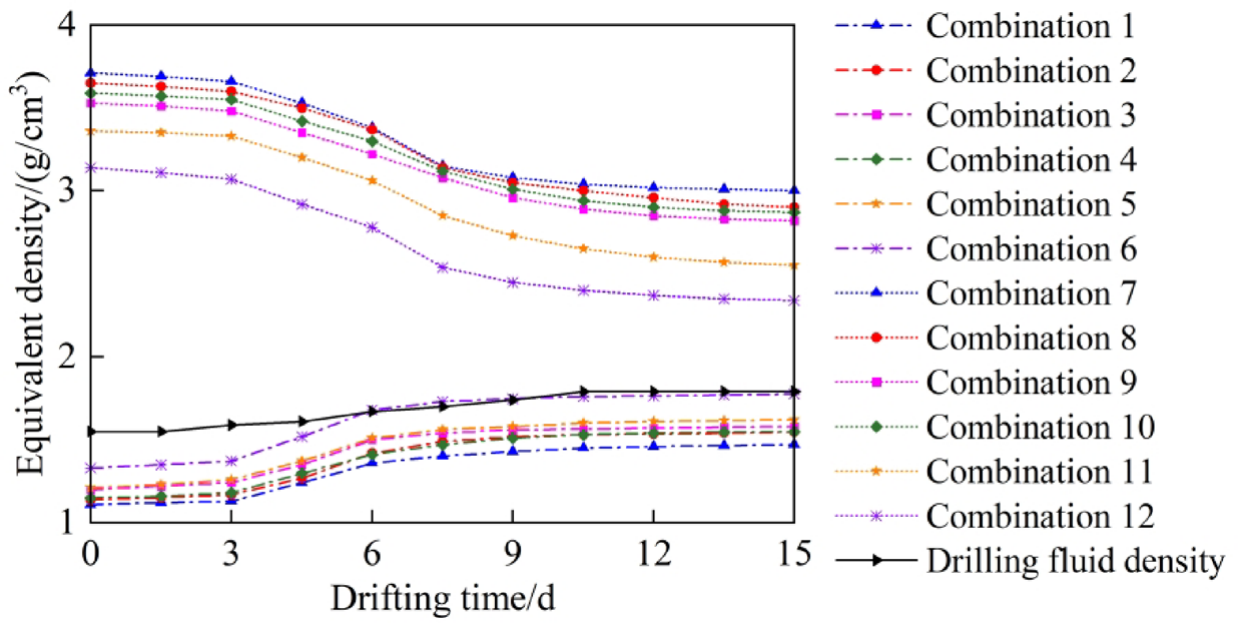
Example analysis results of fluctuating pressure-hydration wellbore stability model.

According to the model constructed in this study, the factors influencing the collapse pressure and fracture pressure of the wellbore during the movement of the drill string include weak structural planes, hydration effects, and fluctuation pressure. Based on different conditions of these influencing factors, 12 combinations were configured, as shown in Table 2. Using the model developed in this study, we compared the predicted equivalent densities of collapse pressure and fracture pressure under various conditions during drilling, as shown in Fig. 6. Under homogeneous conditions without considering hydration and fluctuating pressure, the calculated equivalent densities for collapse pressure range from 1.11 to 1.47 g/cm^3^, and for fracture pressure from 3.00 to 3.71 g/cm^3^. Under homogeneous conditions with hydration considered, the calculated equivalent densities for collapse pressure range from 1.14 to 1.55g/cm^3^, and for fracture pressure from 2.90 to 3.65 g/cm^3^. Under homogeneous conditions considering both hydration and fluctuating pressure, the calculated equivalent densities for collapse pressure range from 1.20 to 1.58 g/cm^3^, and for fracture pressure from 2.82 to 3.53 g/cm^3^. Considering weak planes but excluding hydration and fluctuating pressure, the predicted collapse pressure equivalent densities range from 1.15 to 1.55 g/cm^3^, and fracture pressure equivalent densities from 2.87 to 3.59 g/cm^3^. Considering weak planes and hydration but ignoring fluctuating pressure, the predicted collapse pressure equivalent densities range from 1.21 to 1.62 g/cm^3^, and fracture pressure equivalent densities from 2.55 to 3.36 g/cm^3^. With weak planes, hydration effects, and fluctuating pressure all considered, the collapse pressure equivalent densities are predicted to range from 1.33 to 1.78 g/cm^3^, and fracture pressure equivalent densities from 2.34 to 3.14 g/cm^3^. Under actual field conditions, with an initial drilling fluid density of 1.55 g/cm3, significant wellbore collapse occurred. Despite continuous increases in the drilling fluid density, it remained below the equivalent density of collapse pressure. Only when the drilling fluid density was increased to 1.79 g/cm3 did the wellbore collapse significantly improve. The following conclusions can be drawn from the analysis: (1) Under homogeneous conditions, the collapse pressure is significantly lower, while the fracture pressure is significantly higher. (2) When weak planes and hydration effects are considered but fluctuating pressure is neglected, the predicted collapse and fracture pressures show better alignment with actual field conditions compared to homogeneous conditions. (3) When considering weak planes, hydration effects, and fluctuating pressure simultaneously, the predicted collapse and fracture pressures show the best agreement with actual field conditions, confirming the accuracy of the model. (4) By comparing the actual field drilling fluid density with the predicted equivalent densities of collapse and fracture pressures in this study, it was found that the safe density window gradually narrows as the construction time increases. Therefore, to ensure that the drilling fluid density is better suited for downhole operations, adjustments to the drilling fluid density should be made on time. This conclusion also provides important reference data for drilling fluid design in other wells.

**Table 2:** Condition configuration combination table.

Combinations	Output parameters	Conditions
Combination 1	Collapse pressure	Homogeneous condition
Combination 2		Homogeneous condition + Hydration
Combination 3		Homogeneous condition + Hydration + Fluctuating Pressure
Combination 4		Weak plane condition
Combination 5		Weak plane condition + Hydration
Combination 6		Weak plane condition + Hydration + Fluctuating Pressure
Combination 7	Fracture pressure	Homogeneous condition
Combination 8		Homogeneous condition + Hydration
Combination 9		Homogeneous condition + Hydration + Fluctuating Pressure
Combination 10		Weak plane condition
Combination 11		Weak plane condition + Hydration
Combination 12		Weak plane condition + Hydration + Fluctuating Pressure

### Mechanical response law of collapse instability

#### Distribution law of collapse pressure

**Fig. 7:**
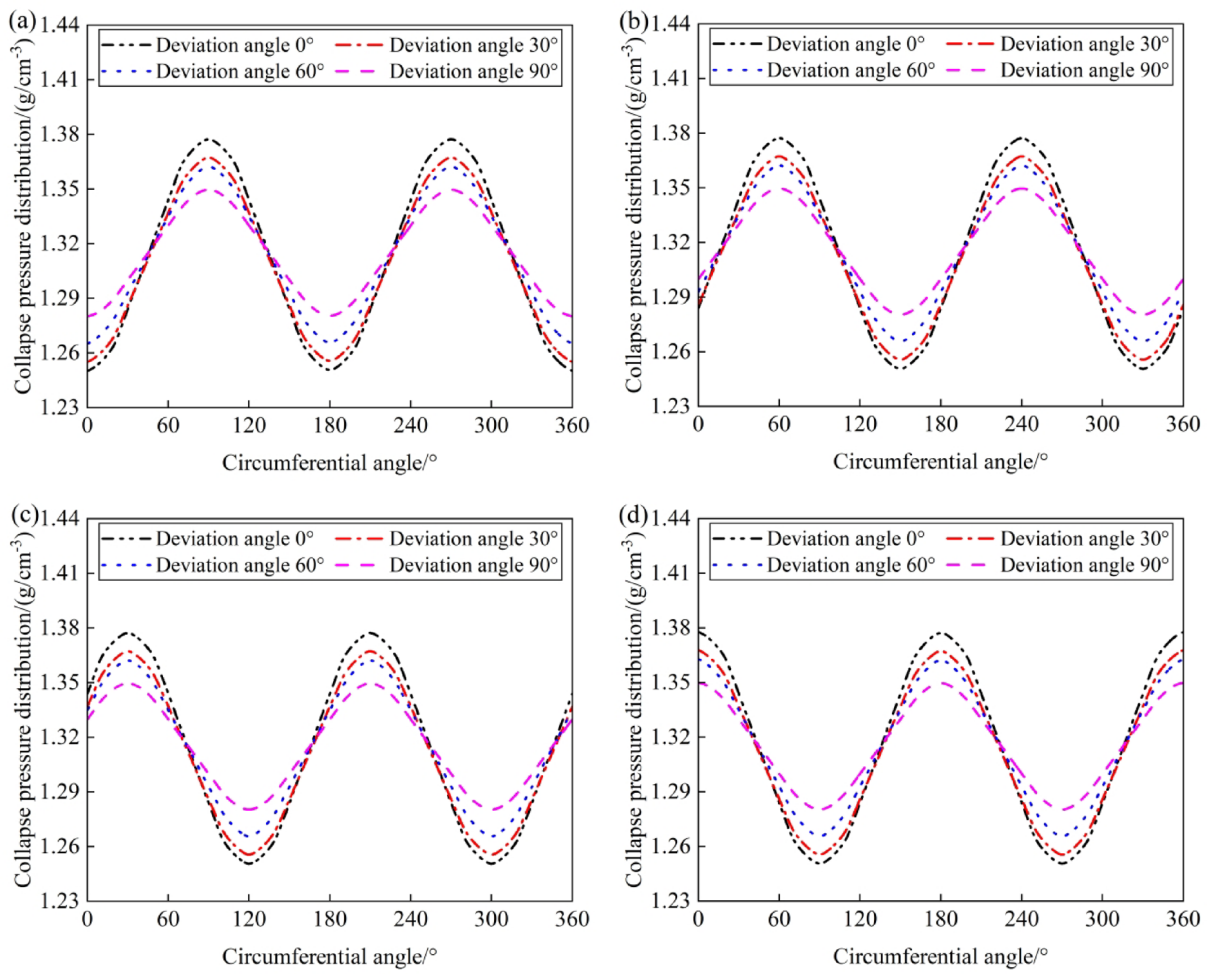
Variation of collapse pressure with circumferential angle and deviation angle: (**a**) azimuth angle 0°; (**b**) azimuth angle 30°; (**c**) azimuth angle 60°; (**d**) azimuth angle 90°.

The variation in collapse pressures with different wellbore angles at inclinations of 0°, 30°, 60°, and 90°, and azimuths of 0°, 30°, 60°, and 90°, is shown in Fig. 7, and is primarily due to the interaction between the well trajectory and the principal stresses in the formation. This interaction dictates the distribution of collapse pressures, with wellbore angles aligned with maximum horizontal stress showing lower collapse pressures and those aligned with the minimum horizontal stress showing higher collapse pressures. These observations highlight the importance of selecting optimal wellbore orientations to minimize collapse risks, particularly when drilling in formations with significant horizontal stress anisotropy. Specifically, at an azimuth of 0°, where the drilling direction matches the maximum horizontal principal stress, the lowest collapse pressures occur at wellbore angles of 0°, 180°, and 360°, while the highest pressures are at 90° and 270°. At an azimuth of 90°, where the drilling direction is aligned with the minimum horizontal principal stress, the lowest collapse pressures occur at wellbore angles of 90° and 270°, while the highest pressures are at 0°, 180°, and 360°. There exists a critical density for drilling fluid; when the drilling fluid density is below this critical value, the wellbore is more susceptible to shear failure along the direction of the minimum horizontal principal stress. As the inclination of the well increases, the maximum collapse pressures gradually increase, and the minimum collapse pressures gradually decrease. As the azimuth changes from 0° to 90°, the wellbore angles corresponding to the maximum collapse pressures shift from 90° and 270° towards 0°, 180°, and 360°, and those for the minimum collapse pressures shift from 0°, 180°, and 360° towards 90° and 270°.

**Fig. 8:**
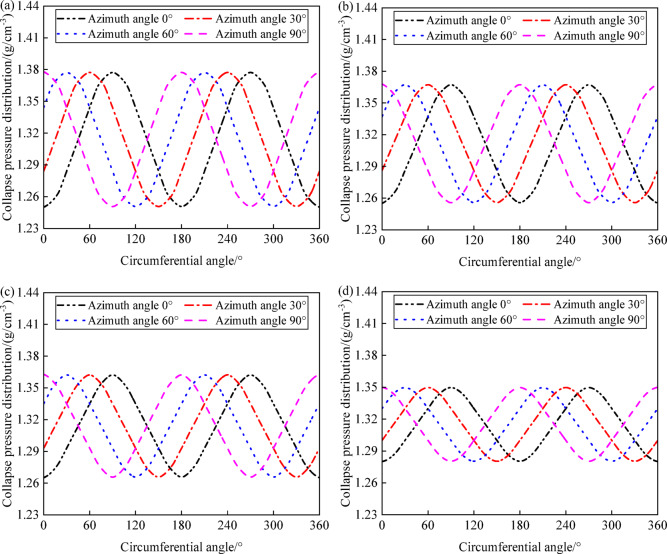
Variation of collapse pressure with circumferential angle and azimuth angle: (**a**) deviation angle 0°; (**b**) deviation angle 30°; (**c**) deviation angle 60°; (**d**) deviation angle 90°.

At well inclinations of 0°, 30°, 60°, and 90°, the patterns of collapse pressures under various azimuths (0°, 30°, 60°, and 90°) with respect to wellbore angles are shown in Fig. 8. As the well inclination increases, the maximum collapse pressure rises, the minimum collapse pressure decreases, and the difference between the maximum and minimum collapse pressures (collapse pressure range) gradually widens. This behavior is critical for wellbore stability analysis, as it reveals how the well trajectory can significantly influence the collapse risk. Understanding this relationship allows for better prediction of collapse pressure ranges, contributing to the safe design of drilling operations, especially in complex geologies with varying stress orientations. When drilling in the direction of the maximum horizontal principal stress, special attention should be paid to the collapse of the wellbore wall at wellbore angles of 90° and 270° in horizontal well sections. When drilling in the direction of the minimum horizontal principal stress, special attention should be paid to the collapse of the wellbore wall at wellbore angles of 0°, 180°, and 360° in horizontal well sections.

#### Effect of wellbore trajectory on collapse pressure

**Fig. 9:**
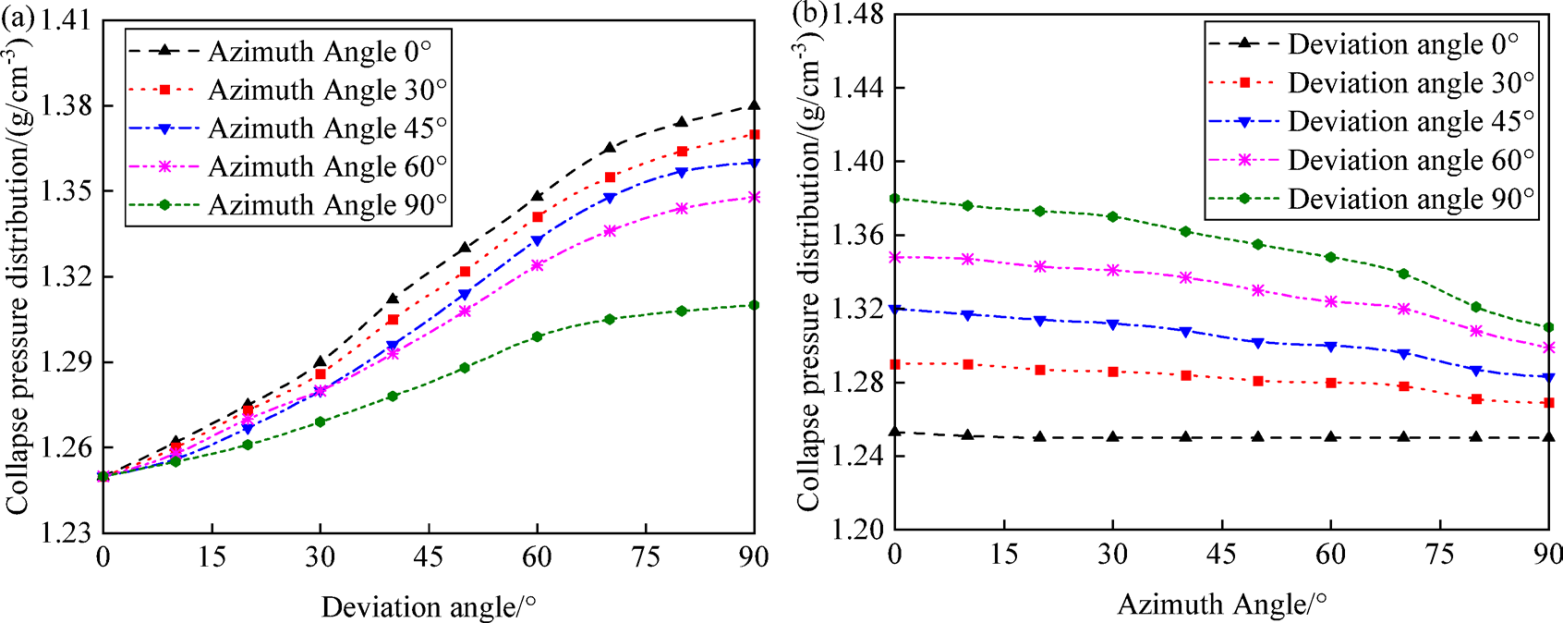
Variation of collapse pressure with well trajectory: (**a**) deviation angle; (**b**) azimuth angle.

Figure 9a illustrates the variation in collapse pressure with well inclination under different azimuths (0°, 30°, 45°, 60°, and 90°). As well inclination increases, collapse pressure also increases, but the rate of increase in collapse pressure decreases with increasing azimuth, indicating that slanted and horizontal wells have weaker wellbore shear resistance and greater instability risk compared to vertical wells. This trend emphasizes the importance of considering well inclination, azimuth, and depth to accurately predict collapse pressures, particularly in inclined or horizontal well sections. In practical wellbore structural design, it is necessary not only to consider the effects of well inclination, azimuth, and depth on wellbore collapse but also to pay attention to the distribution of collapse pressures at different wellbore angles.

Figure 9b shows the variation in collapse pressure with azimuth under different well inclinations (0°, 30°, 45°, 60°, and 90°). When the well inclination is 0°, the collapse pressure is relatively low and remains nearly constant across different azimuths. When the well inclination is 30°, 45°, 60°, and 90°, collapse pressure decreases as azimuth increases. When the well inclination is 90° and the azimuth is 0°, the collapse pressure is at its maximum, making it most susceptible to shear failure.

#### Effect of different amounts of weak planes on collapse pressure

**Fig. 10:**
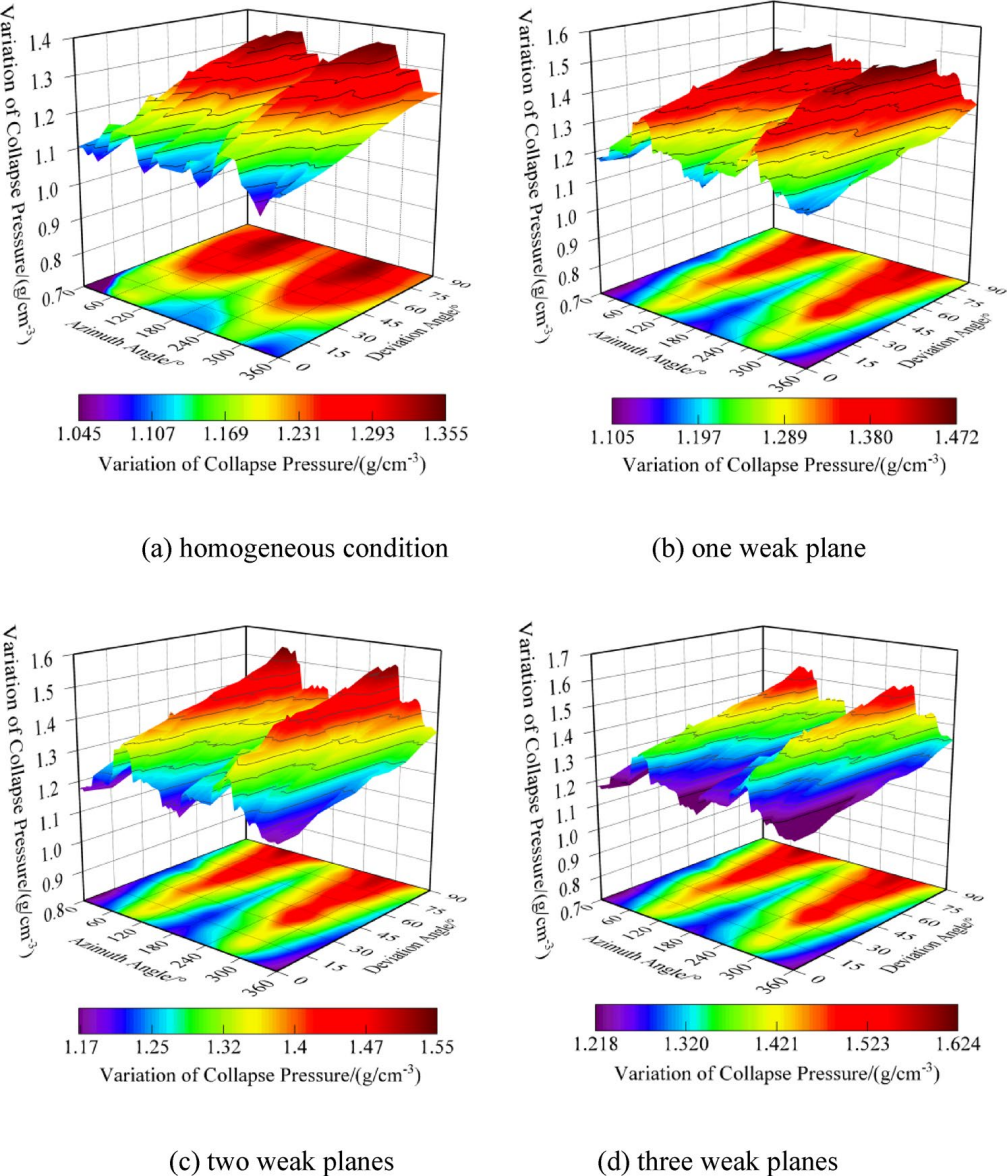
Variation of collapse pressure with different amounts of weak planes.

The frequent interlayering of mudstone-limestone and claystone thin interlayers requires further stability analysis for formations with different numbers of weak planes. As shown in Fig. 10, the distribution of collapse pressure equivalent density for homogeneous formations (excluding claystone interlayers) and under various numbers of weak planes across any wellbore trajectory is illustrated. The collapse pressure calculations for formations with a single set of weak planes show a significant increase compared to homogeneous conditions, with the distribution range rising from 1.045~1.355 g/cm^3^ to 1.105~1.472 g/cm^3^. The ranges of collapse pressure distributions for formations with two and three sets of weak planes are 1.17~1.55 g/cm^3^ and 1.218~1.624 g/cm^3^, respectively. With an increase in the number of weak planes, there is a trend of rising collapse pressure values, narrowing safety margins, and increasing difficulty in tripping. Under homogeneous conditions, collapse pressures are higher in the direction of maximum horizontal stress and lower in the direction of minimum horizontal stress. Considering weak plane failure, the collapse pressures decrease in the direction of maximum horizontal stress and increase in that of minimum horizontal stress, heightening the risk of shear collapse in areas of minimum horizontal stress. The existence of clay thin layers reverses the safe directional orientations for wellbores, which should be carefully considered in stability analyses of mudstone-limestone and claystone thin interlayered formations. Whether under homogeneous or weak plane conditions, as the well inclination increases with a fixed azimuth, the overall trend is for an increase in collapse pressure equivalent density. Additionally, without considering the orientation of weak planes, the collapse pressure distribution shows a clear symmetry under both homogeneous and weak plane conditions. In summary, to ensure smooth drilling operations and reduce the risk of wellbore shear collapse due to tripping, the optimization and selection of wellbore trajectories become increasingly important for formations with multiple weak planes.

#### Effect of hydration time on collapse pressure

**Fig. 11:**
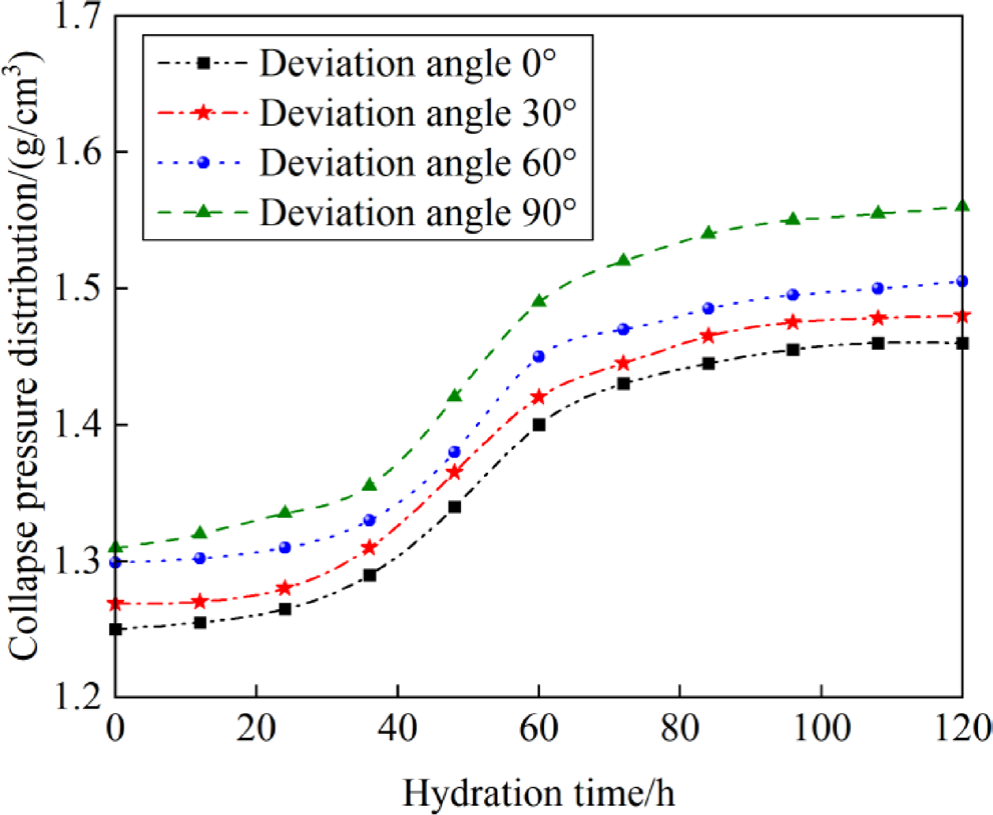
Variation of collapse pressure with hydration time.

Experimental results indicate that claystone, as a weak plane in mudstone-limestone and claystone thin interlayers, exhibits strong hydration characteristics, significantly reducing cohesion and the internal friction angle of the weak planes under the action of drilling fluid hydration. This effect is particularly significant during the tripping process, where the fluctuating pressure causes drilling fluid filtrate to infiltrate newly formed fractures, contacting claystone and further altering the formation density window. As the hydration time increases, the formation’s collapse pressure equivalent density increases, particularly influenced by the well’s inclination. This highlights the importance of controlling hydration time to minimize adverse effects on wellbore stability. During the tripping process, the presence of fluctuating pressure causes drilling fluid filtrate to infiltrate through newly formed fractures, contacting claystone and further altering the formation density window. Figure 11 illustrates the trend in collapse pressure equivalent density over hydration time (0h to 120h) across various well inclinations (0°, 30°, 60°, 90°). The formation’s collapse pressure equivalent density increases with the duration of hydration, significantly influenced by the well’s inclination. During the initial phase of hydration (0h to 36h), the hydration reaction is not yet fully initiated, resulting in a gradual increase in the formation’s collapse pressure equivalent density. During the mid-stage of hydration (36h to 60h), drilling fluid filtrate penetrates deeper into the rock, particularly affecting claystone by accelerating its clay expansion and weakening the rock’s structural strength, which significantly increases collapse pressure. During the later stages of hydration (60h to 120h), the hydration reaction nears saturation, balancing rock expansion and weakening, thereby stabilizing collapse pressures. At 120 hours of hydration, the collapse pressure equivalent density in the vertical section (well inclination at 0°) increases from 1.25 to 1.45 g/cm^3^, and in the horizontal section (well inclination at 90°), it rises from 1.31 to 1.56 g/cm^3^. Compared to vertical wells, slanted and horizontal wells exhibit higher collapse pressure equivalent densities, with drilling fluid hydration time having a greater impact on these wells. It necessitates increasing drilling fluid density and optimizing fluid properties to maintain wellbore stability, thereby increasing the complexity of drilling operations.

#### Effect of tripping speed on collapse pressure

**Fig. 12:**
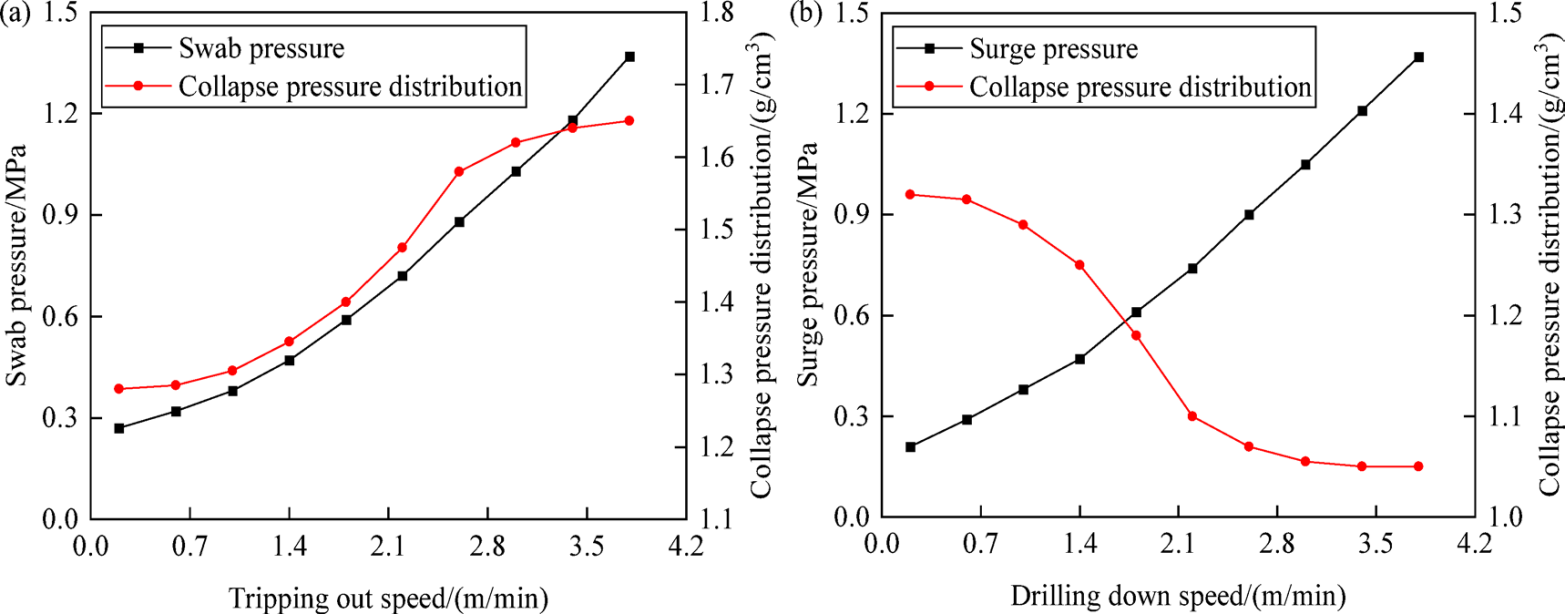
Variation of collapse pressure and fluctuating pressure with tripping speed: (**a**) tripping out speed; (**b**) drilling down speed.

Key factors influencing dynamic pressure are the length of the drill string, properties of the drilling fluid, tripping speed of the drill string, and the geometry of the annulus, with tripping speed being the sole controllable element^39^. Based on this, this section conducts research on the pattern of change in collapse pressure with variations in tripping speed. Figure 12 shows the trends in formation collapse pressure and dynamic pressure as a function of tripping speed. As tripping out speed increases, swab pressure shows a tendency to increase linearly, while the equivalent density of formation collapse pressure exhibits a trend of stability followed by a sharp increase and then stability again, increasing the risk of wellbore instability. As tripping in speed increases, surge pressure likewise trends linearly upward, while the equivalent density of formation collapse pressure stabilizes, sharply decreases, and then stabilizes again, reducing the risk of wellbore instability. Since tripping speed is controllable, there is an optimal tripping speed that can be adjusted to optimize the pressure environment in the wellbore, thus reducing the risk of wellbore collapse and instability.

### Mechanical response law of fracture instability

#### Distribution law of fracture pressure

**Fig. 13:**
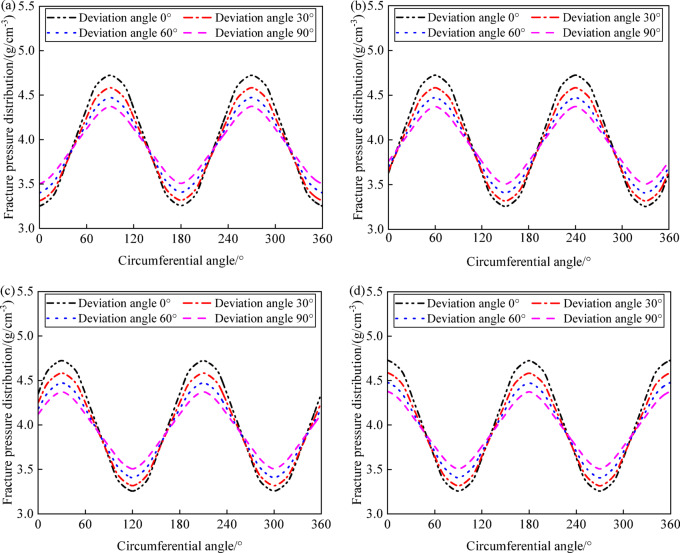
Variation of collapse pressure with circumferential angle and deviation angle: (**a**) azimuth angle 0°; (**b**) azimuth angle 30°; (**c**) azimuth angle 60°; (**d**) azimuth angle 90°.

Figure 13 shows the variation of fracture pressure with wellbore angle under different inclinations (0°, 30°, 60°, and 90°) at wellbore azimuths of 0°, 30°, 60°, and 90°, which is influenced by the relative orientation between wellbore trajectory and the horizontal principal stress. When drilling in the direction of maximum horizontal principal stress, fracture pressure tends to be higher, while drilling in the direction of minimum horizontal principal stress leads to lower fracture pressures. This pattern indicates that careful consideration of azimuth selection is necessary to avoid tensile failure in the wellbore, particularly at wellbore angles where the maximum and minimum fracture pressures occur. Specifically, under different well inclinations, the wellbore fracture pressures are distributed in a periodic pattern with respect to the wellbore angle. At an azimuth of 0°, aligning with the maximum horizontal principal stress, the minimum fracture pressures occur at wellbore angles of 0°, 180°, and 360°, while the maximum pressures are at 90° and 270°. At an azimuth of 90°, corresponding to the minimum horizontal principal stress, the lowest fracture pressures are at wellbore angles of 90° and 270°, while the highest are at 0°, 180°, and 360°. There is a critical value for drilling fluid density; when the drilling fluid density exceeds this threshold, the wellbore is more likely to experience tensile failure along the direction of maximum horizontal principal stress. With the azimuth changing from 0° to 90°, the wellbore angles with the maximum fracture pressures transition from 90° and 270° to 0°, 180°, and 360°, and those with the minimum pressures move from 0°, 180°, and 360° to 90° and 270°.

**Fig. 14:**
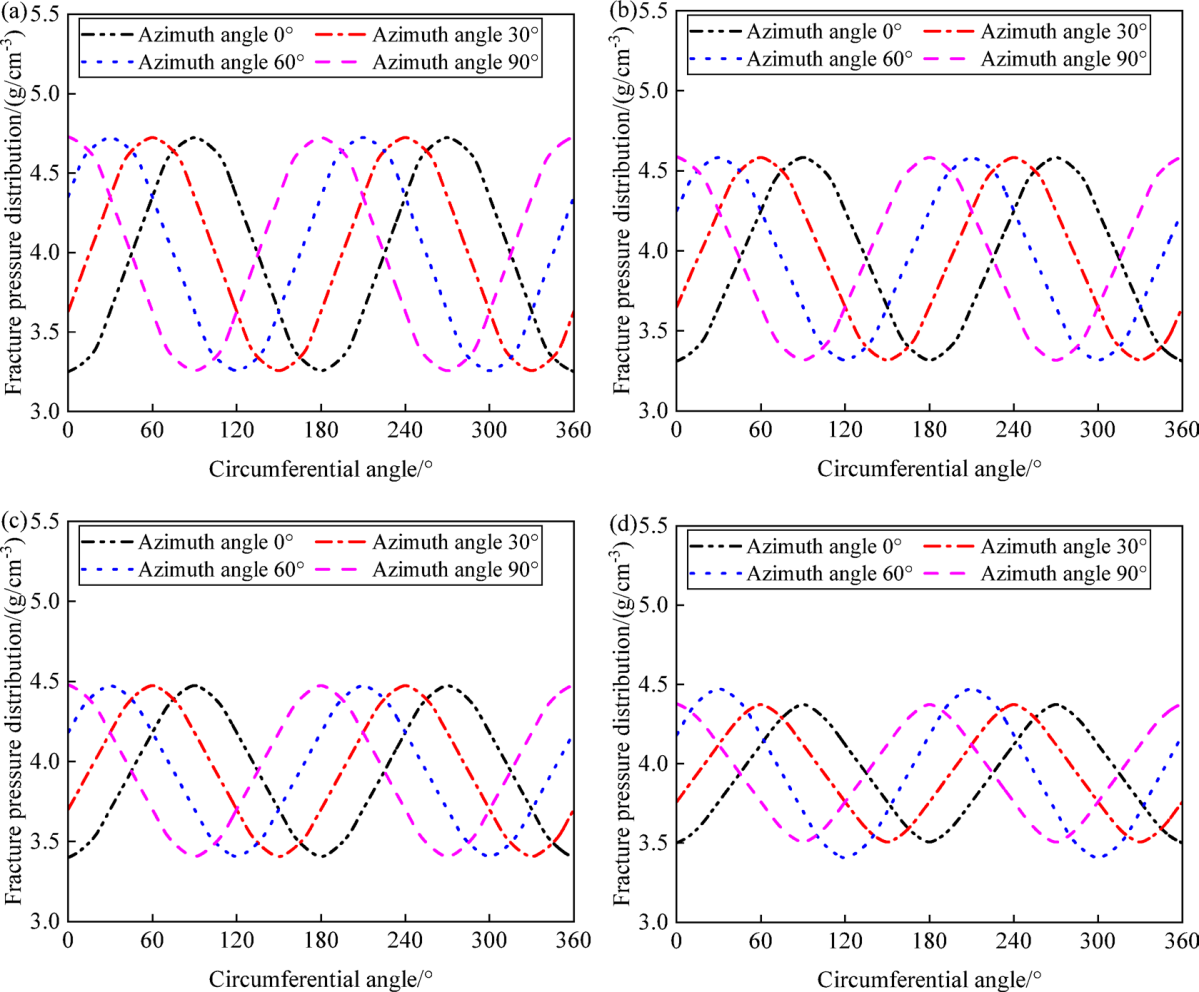
Variation of fracture pressure with circumferential angle and azimuth angle: (**a**) deviation angle 0°; (**b**) deviation angle 30°; (**c**) deviation angle 60°; (**d**) deviation angle 90°.

Figure 14 shows the variation in fracture pressure with wellbore angle at different inclinations (0°, 30°, 60°, and 90°) and azimuths (0°, 30°, 60°, and 90°). As the well inclination increases, the maximum fracture pressure gradually increases, the minimum fracture pressure decreases, and the difference between the maximum and minimum fracture pressures (the fracture pressure range) gradually widens. The wellbore rock is more susceptible to tensile collapse failure in the direction of maximum horizontal principal stress. When drilling in the direction of maximum horizontal principal stress, special attention should be paid to fractures at the wellbore positions of horizontal well segments at angles of 0°, 180°, and 360°. When drilling in the direction of minimum horizontal principal stress, special attention should be paid to fractures at the wellbore positions of horizontal well segments at angles of 90° and 270°.

#### Effect of wellbore trajectory on fracture pressure

**Fig. 15:**
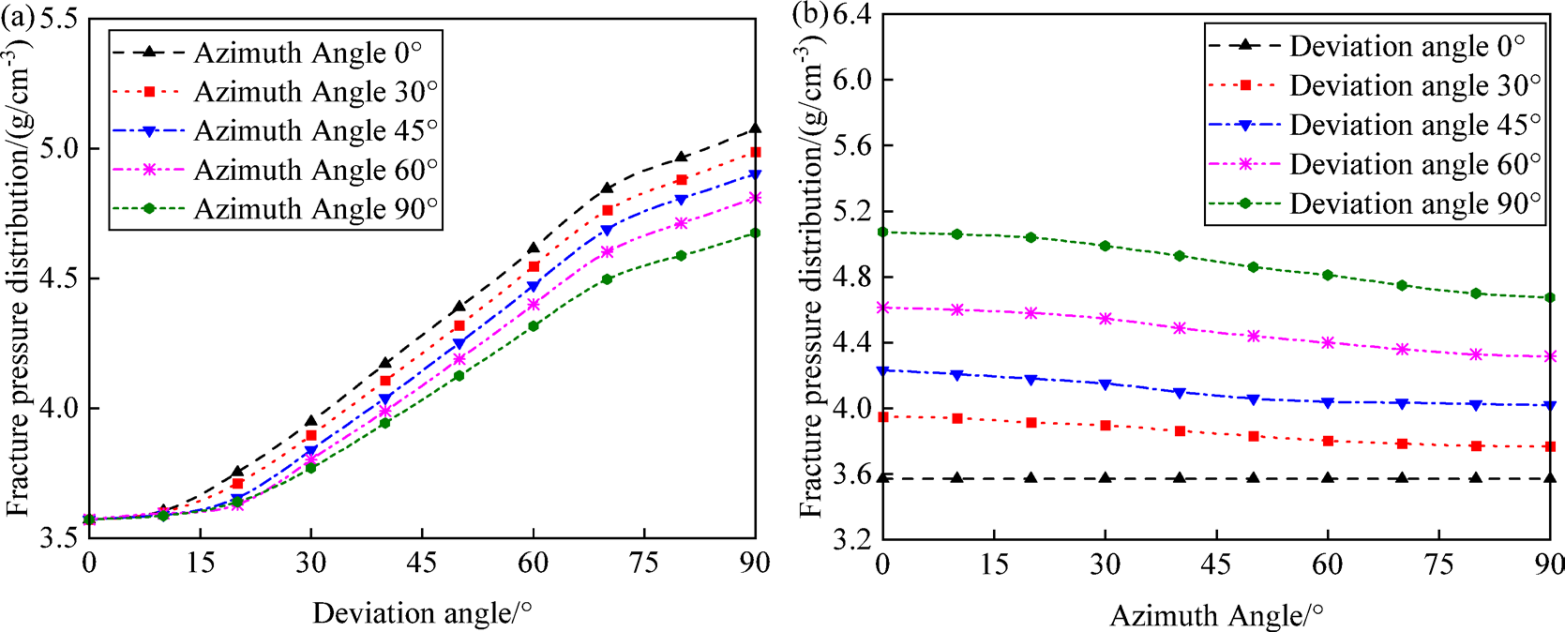
Variation of fracture pressure with well trajectory: (**a**) deviation angle; (**b**) azimuth angle.

Figure 15 shows the variation of fracture pressure with well inclination under different azimuths (0°, 30°, 45°, 60°, and 90°). As the well inclination increases, the fracture pressure also increases, but the rate of increase slows down as the azimuth increases. This indicates that slanted and horizontal wells exhibit weaker wellbore shear resistance and greater instability risk compared to vertical wells. Understanding these trends is crucial for optimizing wellbore trajectories to reduce the risk of tensile failure and enhance overall wellbore stability during drilling operations. Specifically, it is observed that at a well inclination of 0°, the fracture pressure is high and remains relatively constant across different azimuths. When the well inclination is at 30°, 45°, 60°, and 90°, fracture pressure decreases as the azimuth increases. Fracture pressure increases with increasing well inclination, but the rate of increase slows down as the azimuth increases. At a well inclination of 90° and an azimuth of 0°, fracture pressure is at its highest, and the risk of wellbore collapse is at its lowest. At a well inclination of 0° and an azimuth of 90°, fracture pressure is at its lowest, making it most susceptible to tensile failure. This indicates that slanted and horizontal wells have greater resistance to tensile failure and more stable wellbore walls compared to vertical wells. Integrating the patterns of collapse pressure and fracture pressure variations with wellbore trajectory, optimizing the wellbore trajectory, especially choosing the right well inclination, can significantly reduce collapse pressure and increase fracture pressure, thereby enhancing the safety of drilling operations. While the impact of azimuth selection is relatively minor, it is advised to conduct thorough evaluations under specific geological circumstances.

#### Effect of different amounts of weak planes on fracture pressure

**Fig. 16:**
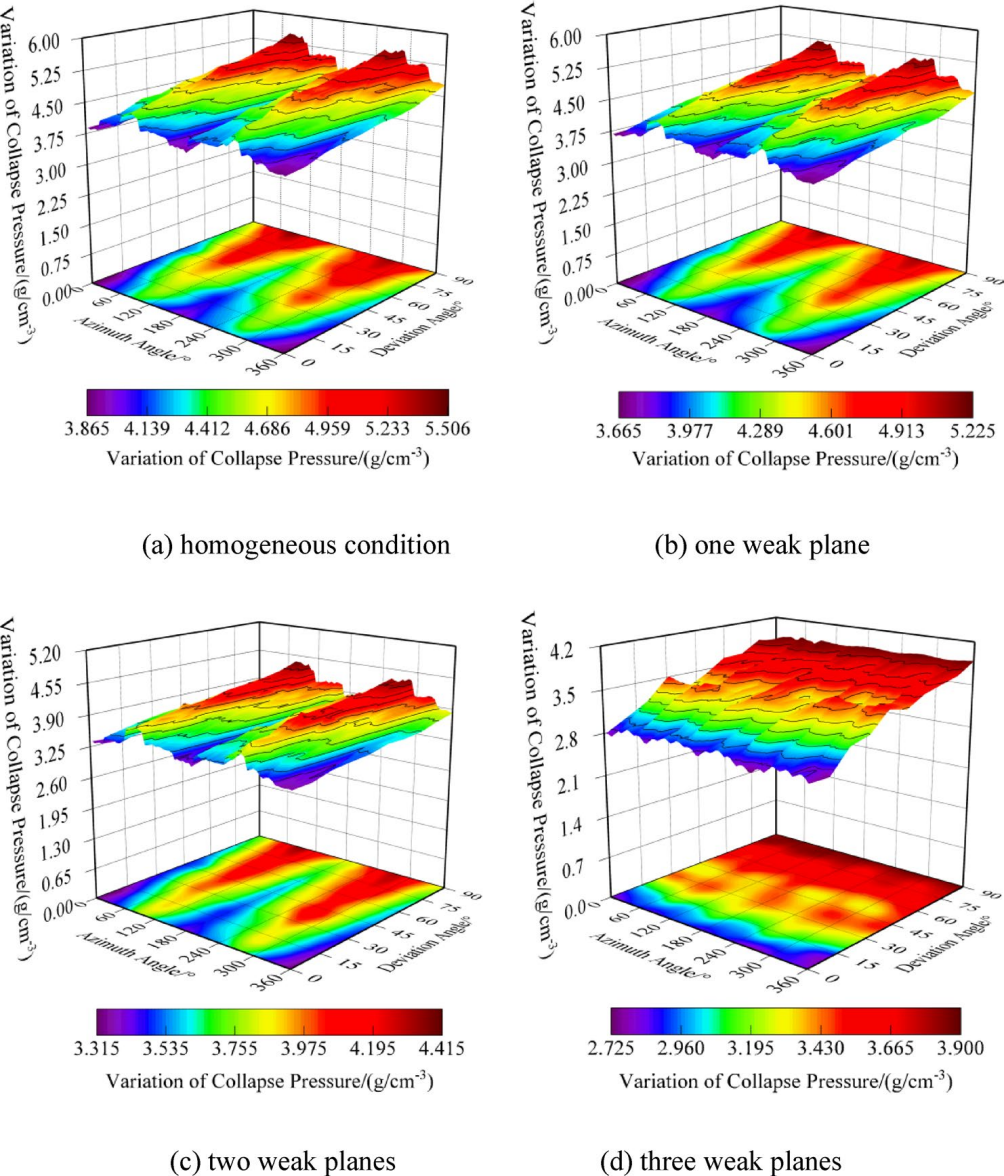
Variation of fracture pressure with different amount of weak planes.

Figure 16 shows the distribution of fracture pressure equivalent densities for homogeneous formations and formations with varying numbers of weak planes under any wellbore trajectory. When only considering the failure of the rock matrix, the fracture pressure equivalent density ranges from 3.869 to 5.506 g/cm^3^, indicating a lower risk of wellbore fracture instability. The ranges of fracture pressure equivalent densities for formations with one, two, and three sets of weak planes are 3.667~5.220g/cm^3^, 3.316~4.412g/cm^3^, and 2.728~3.896g/cm^3^, respectively. When weak planes are present, the overall fracture pressure equivalent density decreases, increasing the risk of tensile fracture in the wellbore. Additionally, as the number of weak planes increases, the fracture pressure equivalent density decreases, the distribution becomes more complex, the safe drilling orientations narrow, and the difficulty of tripping increases. In summary, to ensure the smooth progress of drilling operations and reduce the risk of wellbore tensile fractures caused by tripping, optimizing and selecting the wellbore trajectory is equally important for formations with multiple weak planes.

#### Effect of hydration time on fracture pressure

**Fig. 17:**
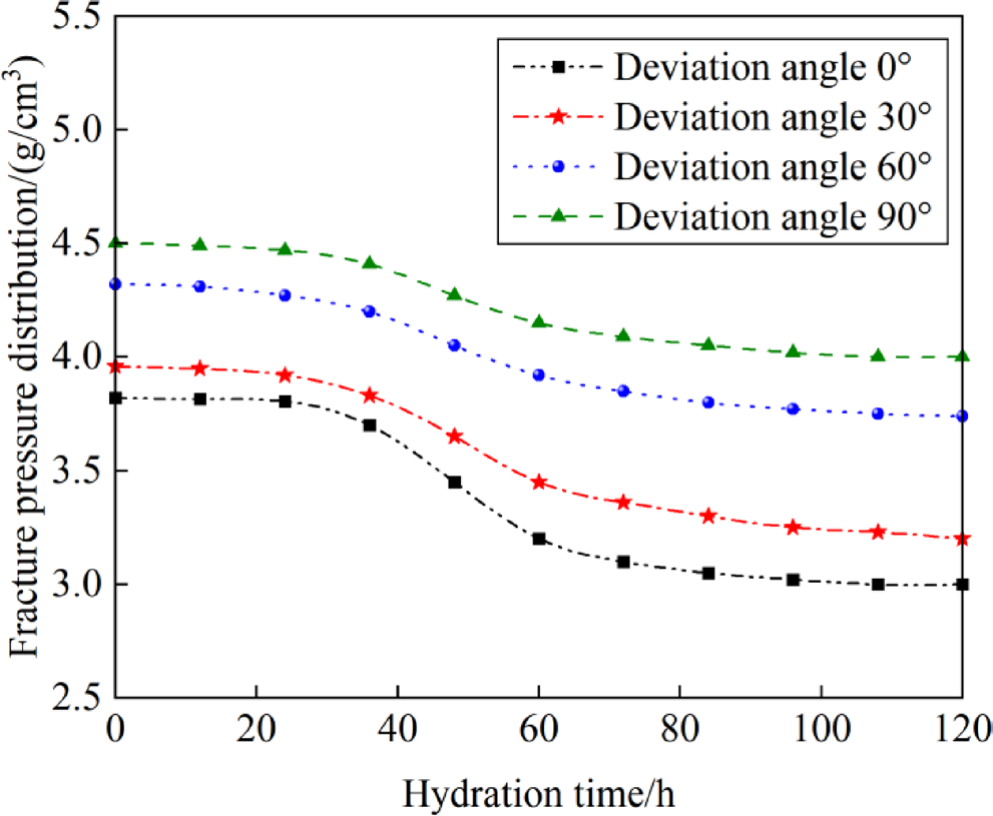
Variation of fracture pressure with hydration time.

Figure 17 displays the trend of fracture pressure equivalent density changes over hydration time (0 to 120 h) at different well inclinations (0°, 30°, 60°, 90°). The fracture pressure equivalent density of the formation decreases with increasing hydration time, significantly influenced by the well inclination. In the initial phase of hydration (0h to 36h), the collapse pressure equivalent density of the formation decreases slowly, then more sharply in the mid-stage (36h to 60h), and stabilizes during the late stage (60h to 120h). At 120 hours of hydration, the fracture pressure equivalent density in the vertical section (well inclination at 0°) decreases from 3.82 to 3.03 g/cm^3^, and in the horizontal section (well inclination at 90°) it decreases from 4.50 to 4.01 g/cm^3^. Vertical wells exhibit lower fracture pressures compared to slanted and horizontal wells, and the effect of drilling fluid hydration time on fracture pressure is more pronounced in vertical wells. Therefore, considering the variation pattern of collapse pressure with hydration time, during drilling operations, prolonged exposure of formations to drilling fluids should be avoided to reduce adverse effects on wellbore stability caused by fluid hydration, while controlling drilling fluid density and optimizing fluid properties to expand the safe density window and maintain wellbore stability.

#### Effect of tripping speed on fracture pressure

**Fig. 18:**
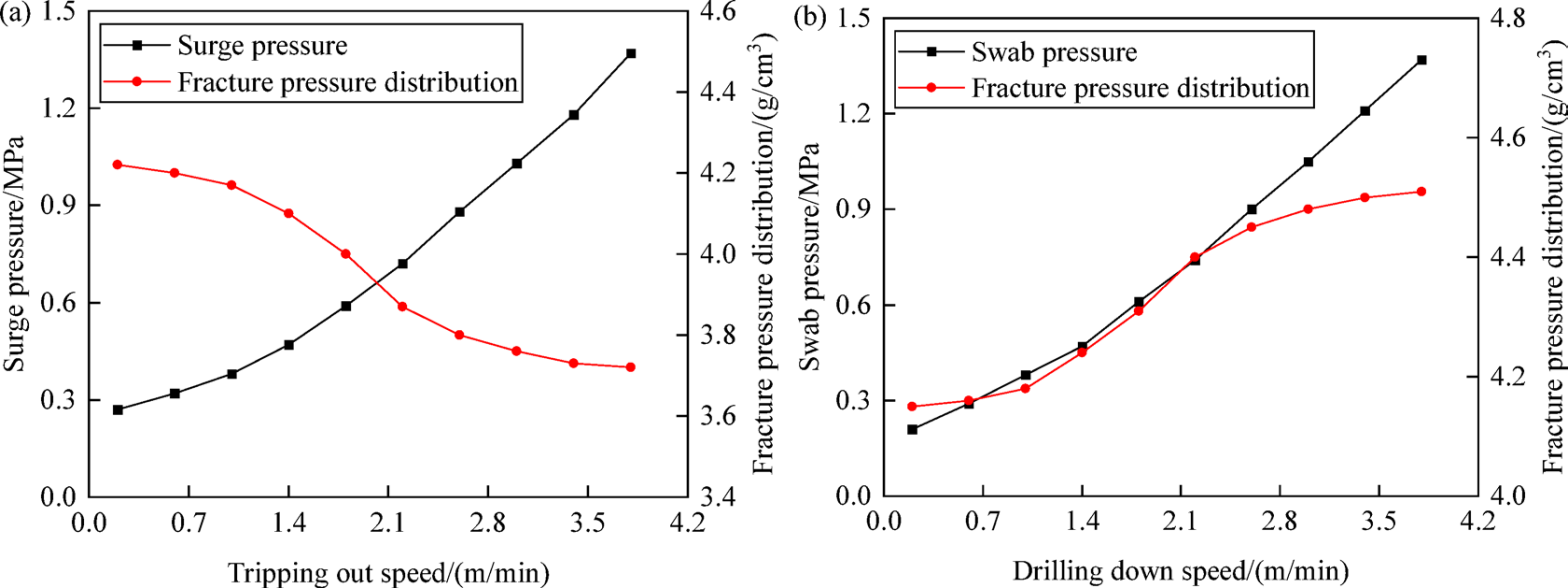
Variation of fracture pressure and fluctuating pressure with tripping out and drilling down speed: (**a**) tripping out speed; (**b**) drilling down speed.

Tripping out speed primarily affects formation collapse instability, while tripping in speed mainly impacts formation tensile fracture instability^40,41^. Figure 18 shows the variation in formation fracture pressure and dynamic pressure with changes in tripping speed. As the tripping out speed increases, swab pressure shows a linear increasing trend, and the equivalent density of formation fracture pressure initially stabilizes, then rapidly increases, and finally stabilizes again, reducing the risk of wellbore instability. As tripping in speed increases, surge pressure grows linearly, while the equivalent density of formation fracture pressure stabilizes, then decreases sharply, and stabilizes again, heightening the risk of wellbore instability. Therefore, to ensure a reasonable and safe density window and reduce the risk of wellbore collapse and fracture instability, it is important to control tripping speeds appropriately during the tripping process, monitor wellbore pressure changes caused by tripping in real-time, and formulate effective drilling operation strategies.

## Conclusion

For tripping operations, based on the mechanical properties of mudstone-limestone thin interlayers under hydration effects, a wellbore stability model for mudstone-limestone thin interlayers under the combined effect of fluctuating pressure and hydration has been developed. The model’s accuracy was validated with field examples, and an analysis of factors affecting wellbore stability under tripping conditions was conducted, leading to the following conclusions:

1. In this research, addressing wellbore instability during tripping operations, tests on the mechanical properties of rocks under hydration effects were conducted. On the foundation of traditional wellbore stability analysis models, variables related to strength damage due to hydration and fluctuating pressure were incorporated. Utilizing principles from damage mechanics, elastic mechanics, and structural face strength theory, and considering the combined impacts of intrinsic damage, weak plane damage, hydration reactions from drilling fluids, and fluctuating pressure from tripping, the study established a stress distribution around the wellbore, formulated criteria for rock shear and tensile failures, and developed a wellbore stability model for mudstone-limestone thin interlayers under the combined influence of fluctuating pressure and hydration.
2. By optimizing the wellbore trajectory, especially choosing the right well inclination, one can significantly reduce collapse pressures and increase fracture pressures, thus improving the safety of tripping operations. Although the choice of azimuth has a smaller impact, it is still recommended to conduct a detailed assessment under specific geological conditions. The presence of weak planes results in increased overall collapse pressures and reduced fracture pressures, leading to narrower safe drilling orientations and increased risks of wellbore instability, which complicates tripping operations.
3. In the process of tripping, it is important to prevent formations from being soaked in drilling fluid for extended periods, reducing the negative impact of fluid hydration on wellbore stability. Simultaneously, controlling the density of the drilling fluid and enhancing its performance are crucial to expanding the safe density window and ensuring wellbore stability. Swab pressures during tripping out primarily impact the distribution of collapse pressures, whereas surge pressures during tripping in predominantly affect the distribution of fracture pressures. To ensure a reasonable and safe density window and reduce the risks of wellbore collapse and fracture instability, it is crucial to control tripping speeds adequately during tripping operations and monitor wellbore pressure changes in real time to devise effective tripping strategies.
4. The practical application of the findings in this study provides valuable guidance for real-world drilling operations, particularly in optimizing drilling fluid density and tripping speed in thin interbedded formations. The wellbore stability model developed in this study has practical significance for wellbore stability control and the design of operational strategies, reducing risks and improving operational efficiency during tripping in challenging formations.
5. The model developed in this study is based on a steady-state method for solving fluctuating pressure. While it offers strong operability and a significant safety margin, it does not reflect the real-time propagation of fluctuating pressure within the wellbore, and its accuracy may not meet the practical requirements of tripping conditions. Future research will focus on developing transient fluctuating pressure models that can simulate dynamic field conditions. In addition, further exploration of the dynamic mechanical response of hydrated weak planes will be crucial to improving the model’s applicability under varying drilling conditions, especially in real-time field operations.
6. The model developed in this study can be adapted to formations with different geological features such as fractured or heterogeneous rock formations. By modifying the weak plane definition to represent fracture networks or anisotropic zones and adjusting parameters like fracture orientation, permeability, and rock heterogeneity, the model can be extended to account for more complex geological settings. Further studies could focus on refining the model to include fracture mechanics and heterogeneity in order to better predict wellbore instability in such formations.

## Data Availability

All data, models, and code generated or used during the study appear in the submitted article.
